# Ccdc113/Ccdc96 complex, a novel regulator of ciliary beating that connects radial spoke 3 to dynein g and the nexin link

**DOI:** 10.1371/journal.pgen.1009388

**Published:** 2021-03-04

**Authors:** Rafał Bazan, Adam Schröfel, Ewa Joachimiak, Martyna Poprzeczko, Gaia Pigino, Dorota Wloga

**Affiliations:** 1 Laboratory of Cytoskeleton and Cilia Biology, Nencki Institute of Experimental Biology of Polish Academy of Sciences, Warsaw, Poland; 2 Max Planck Institute of Molecular Cell Biology and Genetics, Dresden, Germany; 3 Human Technopole, Milan, Italy; UC Denver Anschutz Med Campus, UNITED STATES

## Abstract

Ciliary beating requires the coordinated activity of numerous axonemal complexes. The protein composition and role of radial spokes (RS), nexin links (N-DRC) and dyneins (ODAs and IDAs) is well established. However, how information is transmitted from the central apparatus to the RS and across other ciliary structures remains unclear. Here, we identify a complex comprising the evolutionarily conserved proteins Ccdc96 and Ccdc113, positioned parallel to N-DRC and forming a connection between RS3, dynein g, and N-DRC. Although Ccdc96 and Ccdc113 can be transported to cilia independently, their stable docking and function requires the presence of both proteins. Deletion of either *CCDC113* or *CCDC96* alters cilia beating frequency, amplitude and waveform. We propose that the Ccdc113/Ccdc96 complex transmits signals from RS3 and N-DRC to dynein g and thus regulates its activity and the ciliary beat pattern.

## Author summary

Motile cilia and flagella are evolutionarily conserved microtubule-based cell protrusions with the ability to actively beat. Their coordinated movement propels unicellular organisms, sperm cells and zoospores, or drives a flow of the extracellular fluids in multicellular organisms including humans. Lack or dysfunction of motile cilia cause respiratory diseases that can be accompanied by infertility and laterality defects. The emergence of cilia movement requires coordinated activity of the ciliary complexes, including inner and outer dynein arms and radial spokes. The nexin-dynein regulatory complex (N-DRC) is a main node that coordinates the activity of these ciliary complexes and mediates a transduction of the regulatory signals from radial spokes to axonemal dynein motors. Here we described the identification of a new ciliary complex composed of the two proteins, Ccdc96 and Ccdc113 that extends parallel to the N-DRC and connects radial spoke 3 with N-DRC and dynein g. Knockout of the gene encoding either of these two proteins results in altered pattern of cilia motion. Our data suggest that the Ccdc113/Ccdc96 complex transmits signals from radial spoke 3 and N-DRC to dynein g, and thus further contribute to the understanding of the molecular mechanism(s) behind the regulation of the ciliary beat.

## Introduction

Motile cilia and flagella are microtubule-based cell protrusions that are indispensable for the development and physiology of eukaryotic organisms from a wide range of evolutionary lineages, including humans. The beating of cilia and flagella is regulated by the precise interplay of a large number of components that self-organize to form a complex machine. At the core of this machine is the axoneme, composed of nine outer microtubule doublets and two central microtubules that together serve both as a skeleton for the cilium and a docking site for numerous ciliary complexes [1–15], for review [16]. The main complexes of the outer doublets form a pattern that extends along the entire length of the axoneme with a characteristic 96-nm repeat, called the axonemal unit. In each axonemal unit, there are four outer dynein arms (ODAs) that are generally identical in their protein composition and function, seven inner dynein arms (IDAs) that differ in their architecture and protein subunits, three radial spokes (RSs), and one nexin-dynein regulatory complex (N-DRC). Cryo-electron microscopy analyses of the axoneme have shown that besides the main complexes, the axonemal unit also contains numerous minor structures, mostly of unknown protein composition and function [2,4,10,12,14,15]. The activity of these axonemal complexes has to be strictly coordinated locally within the axonemal unit and globally, both circumferentially and longitudinally along the axoneme, in order for it to be translated into normal ciliary beating [17]. How such coordination is achieved, however, remains largely unclear.

One factor known to play a key role in regulating the activity of ciliary complexes both within the axonemal unit and across the cilium circumference is the N-DRC [17]. This large complex is composed of eleven subunits [18,19] that organize into two well-defined regions: a part named a base plate docked onto the A-tubule of one microtubule doublet and a large linker extending from the base plate to the B-tubule of the adjacent outer doublet. Thus, the N-DRC, besides connecting and coordinating diverse complexes of the 96-nm axonemal unit, also functions as a linker between neighboring microtubule doublets and is fundamental for the integrity of the axoneme [17]. Deletion of different N-DRC subunits causes a range of motility defects, alters flagella waveform and impairs dynein coordination, depending on the targeted subunit [20–24]. Mutations that affect N-DRC subunits can restore some forms of flagella motility in *Chlamydomonas* central apparatus and radial spoke mutants, but do not rescue cell motility [20,21,25]. This suggests that, although the N-DRC complex can influence the activity of dynein arms, a signal from the central apparatus transmitted by radial spokes to dynein arms [26] is required to generate forces that enable cell swimming. How such a signal is transmitted from the central pair, through the radial spokes and the N-DRC to different inner and outer dynein arms is not fully understood.

Analyses in *Chlamydomonas* show that N-DRC makes at least 10 inter- and intra-molecular connections with other complexes within the axonemal unit, some of which are known to be involved in the regulation of the cilium beat [17]. However, which protein(s) build the connections mediating interactions between N-DRC and other axonemal complexes is largely unknown. Here, we describe the identification of a new structure that connects the N-DRC with IDA g and the Fap251-containing arch at the base of RS3 in the ciliate *Tetrahymena thermophila*. We provide evidence that this structure is composed of Ccdc96 and Ccdc113 proteins and is required for normal cilia beating. The existence of this structure further supports the signal transduction hypothesis [26] and sheds new light on the multi-level regulation and coordination of the complex molecular machine that powers the beating of the cilium.

## Results and discussion

### Identification of two candidates to connect the N-DRC with other axonemal complexes in *Tetrahymena*

**Fig 1:**
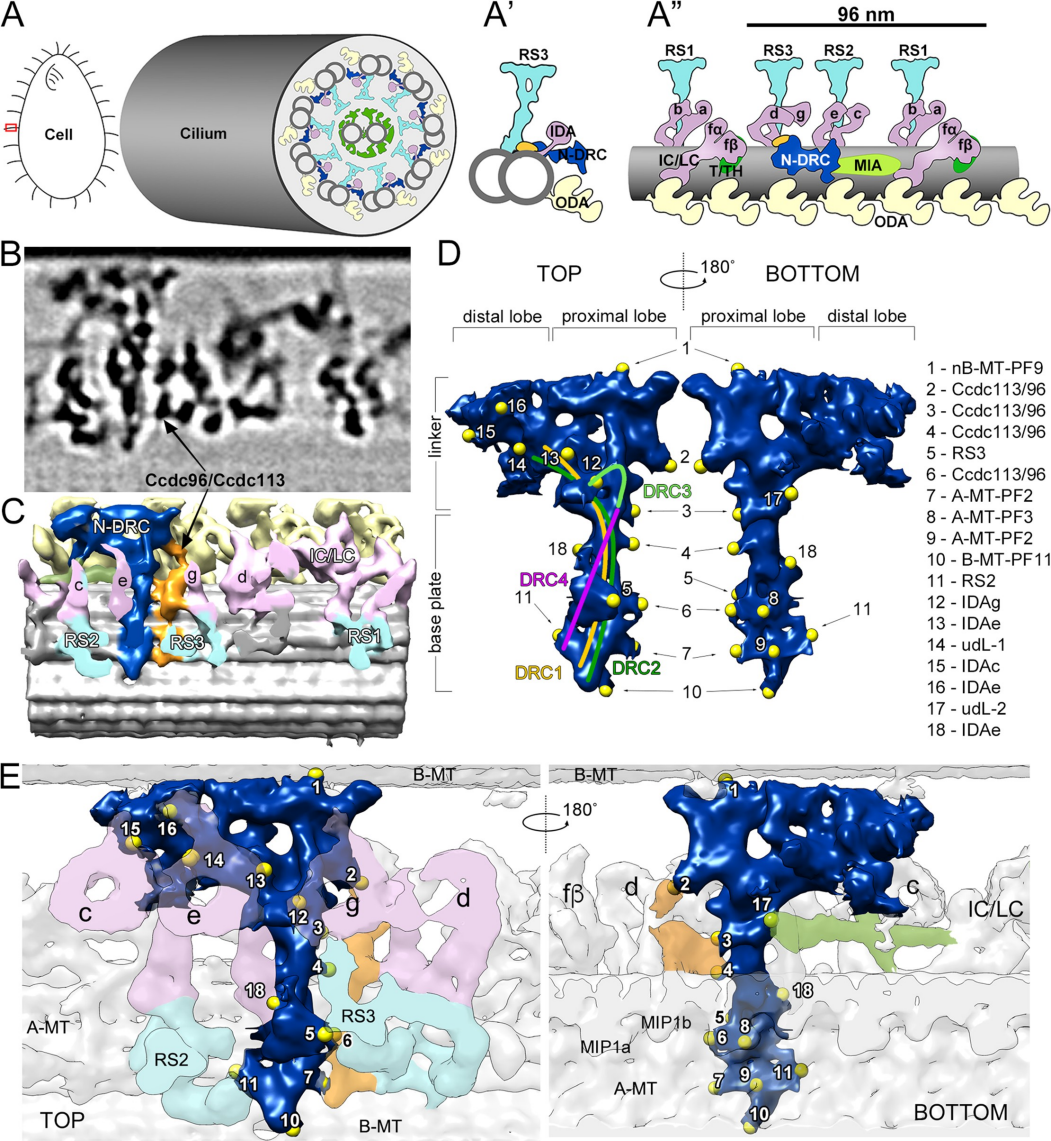
Cryo-ET structure of *Tetrahymena* N-DRC. (A) Schematic representation of a *Tetrahymena* cell and a cross-section of the cilium (marked with a red rectangle on the diagram of *Tetrahymena*) showing its ultrastructural organization. (A’, A”) Schematic representation of a microtubule doublet cross-section (A’) and a schematic of a longitudinal view of a microtubule doublet with the 96-nm axonemal repeat and its main protein complexes (A”): ODAs (yellow), two-headed (f/I1) and single-headed (a, b, c, e, d, g) IDAs (pink), radial spokes (cyan), N-DRC (navy blue), T/TH (dark green), MIA-like complex (light green) and Ccdc96/Ccdc113 complex (orange); (prepared based on cryo-ET images [6,7]). (B) Cryo-tomographic slice through the averaged volume of the wild-type 96-nm repeat showing the position of the N-DRC. (C) Corresponding isosurface rendering of the 96-nm repeat average with the N-DRC colored in navy blue. (D) The segmented N-DRC structure as seen from the central pair complex (left) and from the opposite (~180 degrees rotation) side (right). The yellow spheres represent the individual connections (1–18) to neighboring axonemal components. The putative position of some of the N-DRC proteins (DRC1 yellow, DRC2 green, DRC3 bright green, DRC4 pink) was estimated by comparing our structure with published ones from both *Tetrahymena* and *Chlamydomonas* models [17,24,28,29]. Connections with the Ccdc113/Ccdc96 complex were identified by comparison of the 96 nm repeat structure from WT with the structures of CCDC113-KO and CCDC96-coDel mutant cells. The identification of other axonemal protein complexes that connect with the N-DRC, potentially directly or through yet unidentified proteins, was derived by comparisons of our structure with previously published structures of the 96 nm repeat in different species [4–10,17,24,28,29]. (E) N-DRC (navy blue) connections (yellow spheres) shown in the context of the axoneme, (IDAs (pink), radial spokes (cyan), MIA-like complex (light green), and Ccdc96/Ccdc113 complex (orange). List of connections: nBMT-PF9, protofilament 9 of the neighboring B-tubule; B-MT-PF11, protofilament 11 of the B-tubule; Ccdc113/Ccdc96, Ccdc113/Ccdc96 complex; RS3, radial spoke 3; A-MT-PF2, protofilament 2 of the A-tubule; A-MT-PF3, protofilament 3 of the A-tubule; RS2, radial spoke 2; IDA g (e, c, e), inner dynein arms g (e, c, e, respectively); udL1, undefined linker 1; udL2, unidentified linker 2. Abbreviations: a, b, c, e, d, g–single-headed inner dynein arms; fα, fβ–dynein heavy chains of two-headed inner dynein arm IDA f/I1; IC/LC–intermediate and light chains of the IDA f/I1, MIP1a, MIP1b –microtubule inner proteins 1a and 1b; MIA–modifier of inner arms, N-DRC–nexin-dynein regulatory complex; RS1, RS2, RS3 –radial spokes 1, 2 and 3, T/TH–tether/tetherhead complex.

To provide novel insights into how the N-DRC contributes to the coordination of axonemal complexes activity, we searched for additional, as-yet unidentified N-DRC components or connectors. First, we used cryo-electron tomography (cryo-ET) followed by subtomogram averaging to obtain three dimensional reconstructions of the axonemal units from *Tetrahymena* wild-type cilia and established a more detailed 3D model of the N-DRC structure (Fig 1), which revealed several previously unknown connections with neighboring complexes. Compared to the *Chlamydomonas* N-DRC, for which only one connection to IDA g is known [17] (connection 12; Fig 1D), we observed four additional connections with the inner dynein arms in *Tetrahymena*, one with IDA c (connection 15; Fig 1D) and three with IDA e (connections 13, 16, and 18; Fig 1D). The *Tetrahymena* N-DRC also formed four connections with an as-yet uncharacterized complex that extends parallel to the N-DRC over the A-tubule surface (connections 2–4 and 6; Fig 1D). Only one of these connections (connection 4; Fig 1D) was described previously [17]. We identified new connections between the N-DRC and the radial spokes: besides the previously described connection with the base of RS2 (connection 11; Fig 1D) [17], the base plate protrusion of N-DRC connects to the stalk of RS3 (connection 5; Fig 1D). Connections of the N-DRC base with both tubule A (connections 7–9; Fig 1D) and tubule B (connection 10; Fig 1D) were in agreement with previous findings [17]. The N-DRC linker showed only one connection between the proximal lobe and the B-tubule of the neighboring microtubule doublet (connection 1; Fig 1D), whereas connections are present from both the distal and the proximal lobe in *Chlamydomonas* [17]. In addition to several connections with various IDAs, the distal lobe of the N-DRC also connected to two elongated structures: the first structure extended from the distal lobe to protofilament A5 (connection 14; Fig 1D) and the second structure, which we name as the “96-nm linker” (connection 17; Fig 1D and green dotted line; S1 Fig), ran parallel to the A-tubule from the heavy chain of IDA I1/f to IDA g and IDA d, and contacted the IC/LC of IDA I1/f, the N-DRC, and the MIA-like complex. Thus, this 96-nm linker connects all major components of the 96-nm repeat. Finally, in contrast to *Chlamydomonas*, where an outer-inner dynein (OID) linker connects the N-DRC to ODAs [17,27], we did not find any connection of N-DRC to the outer dynein arms in *Tetrahymena*. Thus, our cryo-ET analysis confirmed the structural similarities between the *Chlamydomonas* and *Tetrahymena* N-DRC, but it also revealed a number of previously undescribed structural differences and connections with other axonemal components. Ultrastructural differences between these two species may result in subtle differences in the N-DRC hub-activity and signal transduction between the axonemal complexes. Thus, structural differences may translate into differences in *Tetrahymena* and *Chlamydomonas* cilia/flagella waveforms and beating.

To identify proteins that connect the N-DRC complex with other axonemal structures, we performed a BioID assay. In the presence of biotin in the culture medium, the expression of a protein with a mutated BirA* ligase attached as a tag results in local (up to 10 nm from BirA*) protein biotinylation [30]. We engineered *Tetrahymena* cells expressing Drc1 (TTHERM_01345750), a subunit of the N-DRC complex, as a C-terminally HA-BirA*-tagged fusion protein under the control of the *DRC1* native promoter (S2 Fig). The Drc1-HA-BirA* localized in cilia (S2A–S2D Fig). Mass spectrometry revealed (besides the N-DRC subunits Drc1, 2, 3, 4 and 7; S1 Table), the presence of two previously uncharacterized proteins, Ccdc113 and Ccdc96, among the ciliary proteins that were specifically biotinylated (S2E Fig) in cells expressing Drc1-HA-BirA* (50 peptides in total and 22 unique peptide sequences for Ccdc96, and 49/22 respectively for Ccdc113). In the same sample, we identified 36/17 peptides of Drc1. These data suggest that Ccdc113 and Ccdc96 proteins are positioned close to the N-DRC and, thus, could connect N-DRC with other axonemal complexes.

### Lack of either Ccdc113 or Ccdc96 impairs cilia motility

Ccdc113 and Ccdc96 are evolutionarily conserved, coiled-coil domain-containing proteins (S3 Fig). So far, these proteins have only been reported to be present in cells assembling primary cilia [31]. In retinal pigment epithelial cells (RPE1), a CCDC96-GFP fusion protein localizes in centrosomes whereas CCDC113-GFP co-localizes with centriolar satellites, and knockdown of *CCDC113* reduces the number of ciliated cells and the length of assembled primary cilia [31]. Survey of a publicly available database (see Material and Methods) revealed that in human and mouse, the CCDC96 and CCDC113 proteins are highly expressed in organs containing cells that assemble motile cilia, such as testis and trachea. Moreover, CCDC96 and CCDC113 are orthologous to the *Chlamydomonas* flagellar proteins FAP184 and FAP263, respectively [32], and our phylogenetic analyses indicate that homologous proteins are also present in other organisms that assemble only motile cilia (S3 Fig). Thus, a function of Ccdc96 and Ccdc113 in motile cilia as potential N-DRC connectors may be conserved in evolution.

**Fig 2:**
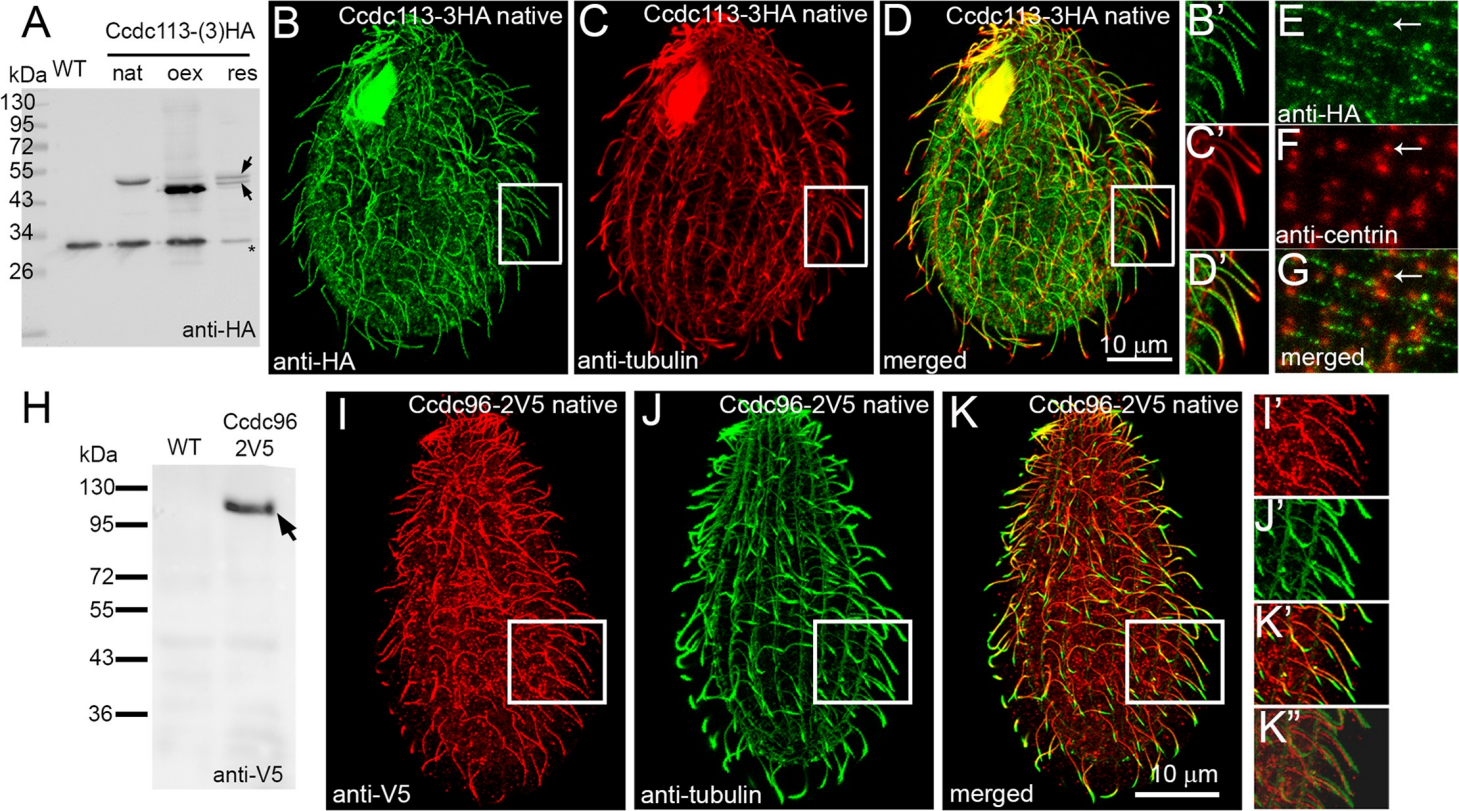
Ccdc113 and Ccdc96 localize in cilia but not at the ciliary tips. (A) Western blot of the ciliary proteins isolated either from wild-type cells (WT) or cells expressing HA-tagged Ccdc113 under the control of a native promoter (native level, nat, with 3HA) or the *MTT1* promoter (overexpression, oex, with single HA), or rescued cells (res, 3HA). Note the presence of two co-migrating bands (arrows), suggesting post-translational modifications of Ccdc113 protein (see also S4A Fig). A star indicates a band which is non-specifically recognized by the secondary antibodies (serving as a loading control). (B-G) Immunofluorescence confocal images of *Tetrahymena* cells expressing Ccdc113-3HA at the native level, double labeled with anti-HA (B, B’ and E) and either anti-α-tubulin (C, C’) or anti-centrin (F) antibodies. (D, D’ and G) Merged images. Note the absence of Ccdc113 at the cilia tips. (B’, C’, D’) The magnified cilia marked with white insets on B, C, and D. Scale bar = 10 μm. (H) Western blot of ciliary proteins isolated from WT cells and cells expressing Ccdc96-2V5 under the control of its native promoter. Arrow indicates the position of the Ccdc96-2V5 protein. (I-K”) Immunofluorescence confocal images of *Tetrahymena* cells expressing Ccdc96-2V5 at the native level, double labeled with anti-V5 (I, I’) and anti-α-tubulin (J, J’) antibodies showing that Ccdc96 protein is distributed along the entire cilium except for the tip. (K-K”) Merged image. (I’, J’, K’, K”) Magnified cilia marked with white inset on I, J and K. (K”) Note the shift in the merged red and green channels enabling better visualization of the region where Ccdc96-2V5 localizes. Scale bar = 10 μm.

*Tetrahymena* Ccdc113 (TTHERM_00312810) is a 43 kDa, likely post-translationally modified, protein (Figs 2A and S4A). These data are in agreement with studies in *Chlamydomonas* suggesting that FAP263 is a phosphoprotein [33]. Regretfully, we were unable to identify the nature of Ccdc113 modification(s). Ccdc96 (TTHERM_00529650) is a larger ~94 kDa protein with poorly conserved N-terminal part but highly conserved C-terminal end (S3 Fig).

When expressed under the control of a native promoter, Ccdc113-3HA localized along the entire cilium, with the exception of the ciliary tip. A limited amount was also observed near the basal bodies (Fig 2B–2G). Similar ciliary localization was observed when Ccdc96-2V5 was expressed under the control of its native promoter (Fig 2H–2K”). Domain analysis of Ccdc96 revealed that the less-conserved N-terminal fragment, HA-Ccdc96 M1-I431, accumulated within the cell body whereas the highly conserved C-terminal part, HA-Ccdc96 A370-Y794, was sufficient for ciliary localization (S5A–S5D Fig).

**Fig 3:**
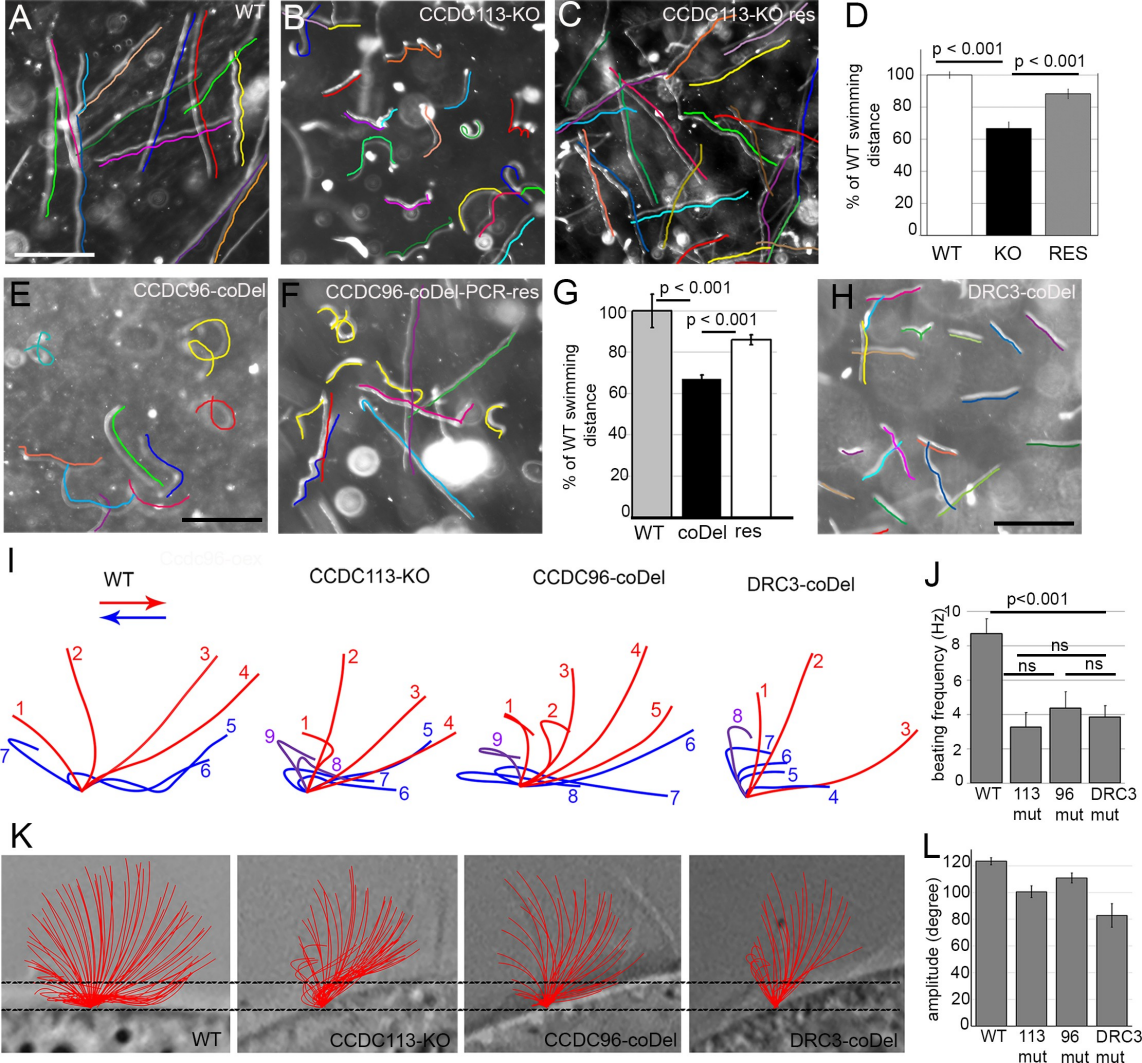
Ccdc113 and Ccdc96 are required for normal ciliary beating. (A-C) Swimming paths of (A) WT, (B) *CCDC113-KO* and (C) *CCDC113-KO* rescued cells recorded for 3.2 s using a video camera. The trajectories are indicated by colored lines; note that the drawn lines were shifted to be positioned parallel to the cell paths and make the original paths visible. Dots are most likely immotile dividing cells. Very short trajectories may represent cells that slowed down in order to change the direction of swimming. Bar = 400 μm. (D) Graph representing the average distance swum by WT, *CCDC113-KO* mutant (KO) and *CCDC113-KO* rescued (res) cells expressing Ccdc113-3HA, normalized to WT values. Error bars represent standard error. (E-F) Trajectories of swimming *CCDC96-coDel* mutant (E) and *CCDC96-coDel* rescued cells (F) recorded for 3.2 s using a video camera. Trajectories are marked with colored lines. Note that no selection pressure to increase the number of wild-type copies of *CCDC96* was applied in rescued mutant and thus some cells have more copies and swam almost as WT cells while others (with low copy number) swam similarly to mutant cells. Bar = 400 μm. (G) Graph representing the average distance swum by WT, *CCDC96-coDel* mutants (coDel) and *CCDC96-coDel* rescued (res) cells normalized to the WT value. Error bars represent standard error. (H) Trajectories of swimming *DRC3-coDel* mutant cells marked with colored lines. Note that *DRC3-coDel* trajectories are straight while those of *CCDC113-KO* and *CCDC96-coDel* mutants are wavy and kinky, suggesting frequent changes in swimming direction. Bar = 400 μm. (I) Drawings representing examples of the observed subsequent positions of a cilium of WT and mutant cells (*CCDC113-KO*, *CCDC96-coDel*, *DRC3-coDel*) during the power (red) and recovery (blue) stroke. The position of the cilium marked in purple represents a cilium that is still bending (as in the recovery stroke) but already lifting up (as in the power stroke). Drawings were prepared using the individual frames extracted from digitized videos of WT and mutant *Tetrahymena*. Traced cilia were positioned at the middle dorsal region of the cell. (J) Graph representing cilia beating frequency of WT and mutant cells. T-test denotes P value < 0.001. (K) The analyses of the ciliary amplitude. The schematic representation of all recorded consecutive positions of the cilium during the power and recovery stroke. The amplitude was measured as the angle between two most angled positions of the cilium (calculated as the angle of the triangle marked by the intersection points of the cilium with the measuring line and cilium base. (L) Graph representing cilia beating amplitude of WT and mutant cells. T-test denotes P value < 0.001 for *CCDC113-KO* and *DRC3-coDel*, and 0.05 for *CCDC96-coDel*. Numerical data are in S10 Table.

To investigate the role of both proteins in motile cilia, we engineered knock outs in *Tetrahymena* cells. Cells of two independently obtained clones lacking the *CCDC113* gene (S4B and S4B’ Fig) assembled cilia of similar length to wild-type cells (S4C–S4E Fig) but their swimming behavior was altered (Fig 3A, 3B and 3D). The swimming speed of the *CCDC113-KO* mutant (200 +/– 8 μm/sec [standard error], n = 125) was reduced compared to wild-type (300 +/– 4 μm/sec, n = 80), and mutants travelled approximately 60% of the distance covered by wild-type cells (Fig 3D). Moreover, in contrast to the straight swimming paths of wild-type cells, the trajectories of *CCDC113-KO* mutants were frequently wavy and kinky (Fig 3A and 3B).

Cell proliferation and phagocytosis, two other processes which depend upon correct cilium function, were also slowed down in *CCDC113-KO* cells (S4F and S4G Fig). Beating of *Tetrahymena* cilia generates a rotational movement of the daughter cells which contributes to the breakage of a connecting bridge during the final stage of cytokinesis [34]. Consistent with this, the *CCDC113-KO* mutants divide slower (S4F Fig) and the calculated doubling time was approximately an hour longer than that of wild-type cells (wild-type– 2.1 h, *CCDC113-KO*– 3.0 h). The synchronous movement of cilia that surround the oral apparatus directs food particles to the oral cavity where food vacuoles are formed [35]. Addition of India ink to the culture medium allows tracking of food vacuoles and estimation of the rate of phagocytosis [36]. On average, during 10 min, *CCDC113-KO* cells form only 3.9 vacuoles (n = 300) whereas wild-type cells have 5.6 vacuoles (n = 300 cells) (S4G Fig). Expression of Ccdc113-3HA from the non-essential *BTU2* locus in *CCDC113-KO* cells (last row; Fig 2A) restored normal swimming pattern and led to recovery of swimming velocity to ~88% of wild-type (265 +/– 4 μm/sec, n = 40) (Fig 3C and 3D). The rates of cell proliferation (2.4 h) and phagocytosis (5.7 vacuoles [n = 300] over 10 min) of the rescued cells were only slightly below those observed in wild type (S4F and S4G Fig). Thus, Ccdc113 appears to be required for normal cilia function.

Deletion of *CCDC96* (five clones obtained; S5E and S5F Fig) by targeted gene disruption in macronuclei (coDel, [37]) caused a similar phenotype to the *CCDC113* knockout: the length of cilia was not affected (S5G Fig) but cell swimming (Fig 3E and 3G), proliferation and phagocytosis rates (S5H and S5I Fig) were reduced. Compared to wild-type cells, which swam with a velocity of 311 +/– 7 μm/sec, (n = 40), the velocity of *CCDC96-coDel* mutants was reduced to 2/3 (212 +/– 4 μm/sec, n = 60) (Fig 3E and 3G). Transformation of *CCDC96*-*coDel* cells with a 3kb DNA fragment containing part of the 5’UTR and a *CCDC96* coding region separated by the HA tag, restored cell velocity (274 +/– 5 μm/sec [~88% of wild-type cells], n = 30) (Fig 3F and 3G). Thus, Ccdc96 is also required for normal cilia function.

*Tetrahymena* cilia beat synchronously, with distinguishable power and recovery strokes. Analysis of the ciliary beating pattern using high-speed video recording revealed that a lack of Ccdc113 resulted in an abnormal ciliary waveform and reduced amplitude and beating frequency (Figs 3I–3L and S6 and S1 and S2 Videos). In wild-type cells, the cilium marks an angle of nearly 180^o^ in a plane perpendicular to the cell surface during the power stroke (in red; Fig 3I) and returns to the initial position moving parallel to the cell surface during the recovery phase (in blue; Fig 3I). In *CCDC113-KO* cells, the angle marked by a beating cilium was smaller and the cilium frequently leaned toward the posterior end of the cell (Figs 3I, 3K and 3L and S6). Moreover, cilia of wild-type cells are straight during the power stroke, whereas they bend during the recovery stroke. The curvature shifts along the cilium length from its base to the tip as the cilium changes its position while moving parallel to the cell surface (Fig 3I and S1 Video). Cilia lacking Ccdc113 were also straight during the power stroke, but during the recovery stroke, the curvature shift was delayed and still in progress when the cilium came back to the upright position to start the next power stroke (positions 8, 9 and 1; Fig 3I and S2 Video). The ciliary beat pattern in *CCDC96-coDel* cells was similar to that of *CCDC113-KO* cells (Figs 3I–3L and S6 and S3 Video), although we observed some alterations in the waveform and amplitude in the subsequent beating cycles of the same cilium. Thus, Ccdc96 and Ccdc113 are required for normal ciliary beating.

### Ccdc96 and Ccdc113 connect the base of RS3 to the tail of IDA g and the N-DRC

**Fig 4:**
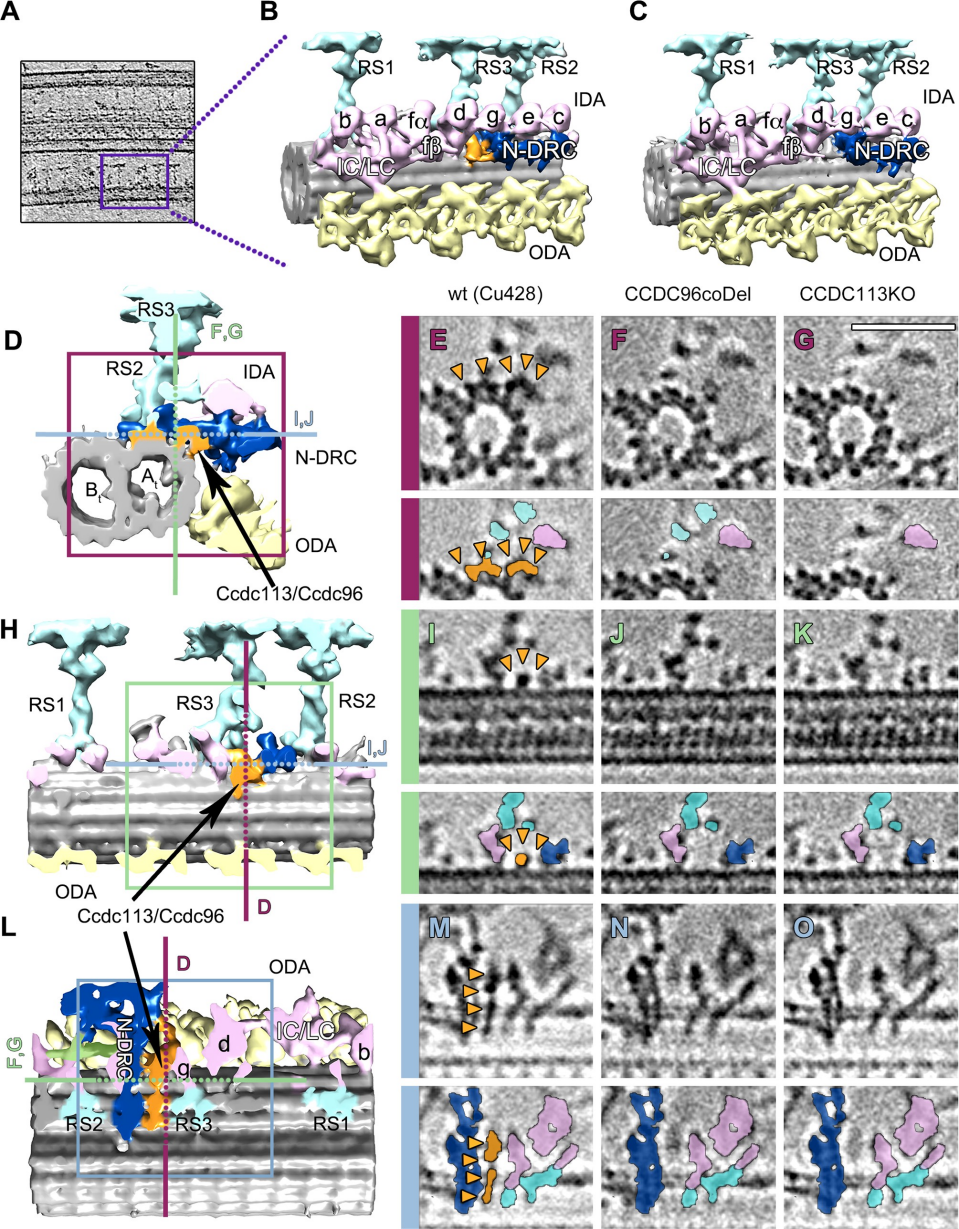
Ccdc113 and Ccdc96 form a complex positioned between N-DRC, RS3, and IDA g. (A) Slice through a subvolume extracted from a filtered tomogram showing the 96-nm repeat (square). (B) 3D isosurface rendering of 96-nm repeat average obtained from wild-type *Tetrahymena* axonemes. (C) 3D isosurface rendering of 96-nm repeat average from *CCDC96-coDel* axonemes. (D, H, L) show the isosurface rendering structure of the Ccdc96/Ccdc113 complex (in orange) in wild-type axonemes. (D) Cross-sectional view. (H) Longitudinal view from the position of the neighboring MTd. (L) Longitudinal view from the position of the central pair (E, I, M) show tomographic slices through D, H, and L, respectively, at the positions indicated by the magenta, green and blue squared and lines in D, H, and L. (F, J, N) show the corresponding tomographic slices through the reconstruction of the *CCDC96-coDel* mutant, and the absence of the Ccdc96/Ccdc113 complex density. (G, K, O) show the corresponding tomographic slices through the reconstruction of the *CCDC113-KO* mutant, and, also in this case, the absence of the Ccdc96/Ccdc113 complex density. (E-G, I-K, M-O) Each tomographic digital section is accompanied by a corresponding segmented copy (bottom panels) with the Ccdc96/Ccdc113 complex colored in orange (and orange arrowheads), the N-DRC in navy blue, the IDA in pink and the radial spokes in cyan. Labels: RS1 to RS3–radial spokes 1 to 3; IDA a, b, c, d, e, g–inner dynein arms a, b, c, d, e, g; IC/LC–intermediate chain/light chain of the inner dynein arm l1/f, fα, fβ-heavy chains α, β of inner dynein l1/f; N-DRC–nexin-dynein regulatory complex; ODA–outer dynein arms. IDAs are named according to [7].

**Fig 5:**
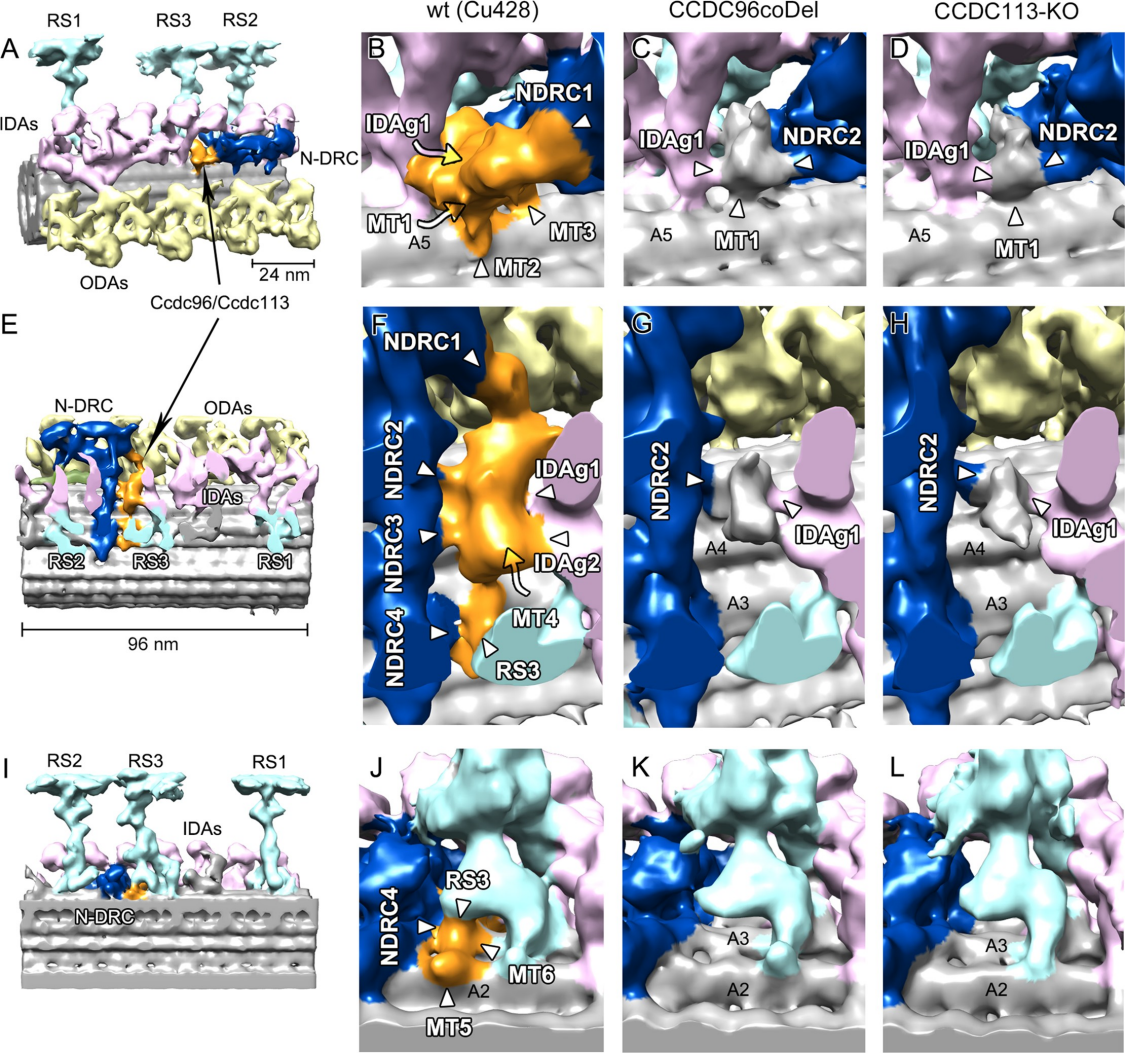
Interactions of the Ccdc96/Ccdc113 complex with neighboring axonemal structures. (A, E, I) Positioning of the Ccdc96/Ccdc113 complex within the 96-nm repeat of WT cilia. (B, F, J) Detailed isosurface rendering of Ccdc96/Ccdc113 complex connections in the WT structure. (B) The wild-type structure of the Ccdc96/Ccdc113 complex shows three connections to protofilament A5 (MT1-MT3), one to the base of IDA g (IDAg1) and two connections to the N-DRC proximal lobe (NDRC1, NDRC2). (C, D) Remnant densities in the corresponding area in both mutant structures show only connections to MT1, NDRC2 and IDAg1. (F) Another view of the wild-type structure of the Ccdc96/Ccdc113 complex shows four connections to the N-DRC (NDRC1-NDRC4), two connections to the base of IDA g (IDAg1, IDAg2), one connection to the protofilament A3 (MT4), and one connection to the base of the radial spoke 3 (RS3). (G, H) Remnant densities in the corresponding area present in both mutant structures show only connections with NDRC2 and IDAg1. (J) View of the base of RS3 in the wild-type structure shows a connection to the basal part of N-DRC (NDRC4), two connections to protofilaments A2 and A3 (MT5, MT6) and a connection to the base of RS3. (K, L) Remnant densities in the corresponding area present in both mutant structures show a completely missing structure in this area.

Traditional TEM analysis of fixed and resin-embedded cilia of *CCDC96-coDel* and *CCDC113-KO* mutants failed to reveal apparent alterations of the axonemal structure. Therefore, we used cryo-ET followed by subtomogram averaging to obtain three-dimensional reconstructions of the axonemal units from wild-type (Figs 4A, 4B, 4D, 4E, 4H, 4I, 4L and 4M and 5A, 5B, 5E, 5F, 5I, and 5J) and mutant cilia (Figs 4C, 4F, 4G, 4J, 4K, 4N, and 4O and 5C, 5D, 5G, 5H, 5K, and 5L), with similar resolutions (35 Å (WT), 35 Å (*CCDC113-KO*) and 34 Å (*CCDC96-coDel*), FSC 0.143 criterion (S7 Fig)). The two independent 3D models of *CCDC113-KO* (Figs 4G, 4K, and 4O and 5D, 5H, and 5L) and *CCDC96-coDel* (Figs 4F, 4J, and 4N and 5C, 5G, and 5K) axonemes revealed strikingly similar morphologies both characterized by the absence of a structure (colored in orange; Figs 4 and 5) that, in wild-type cells (Fig 4D, 4E, 4H, 4I, 4L, and 4M), connects the base of RS3, the tail of the IDA g, and the N-DRC (Figs 4H, 4L, and 5). This structure is composed of a plate that extends across several protofilaments of the A-tubule, from protofilament A2 to A5 (connections MT1 to MT6; Fig 5E, 5F, 5I, and 5J), and a hook-like structure that extends from protofilament A5 to the distal lobe of the N-DRC (connection NDRC1; Fig 5A, 5B, 5E, and 5F). The morphological similarity between the two mutants suggests that Ccdc96 and Ccdc113 might form a complex.

The interpretation of the cryo-ET data was supported by proteomic data showing that both Ccdc96 and Ccdc113 are either dramatically reduced or entirely missing in cilia assembled by *CCDC96-coDel* and *CCDC113-KO* mutants (S2 and S3 Tables). Besides Ccdc96 and Ccdc113 only one protein identified by a significant number of peptides in the wild-type ciliome, Cfap299, was substantially reduced or absent in the mutant ciliomes. However, this approximately 29 kDa protein was not identified among proteins located in the close vicinity of Ccdc96 and Ccdc113 (BioID assay, see below). Thus, most likely, the density missing in *CCDC96-coDel* and *CCDC113-KO* mutants is composed of only two proteins, Ccdc96 and Ccdc113.

In both *CCDC113-KO* and *CCDC96-coDel* mutants, a part of a Fap251-containing arch-like structure at the base of RS3 [38] was missing (Fig 5G, 5H, 5K, and 5L). Thus, the most distal part of the arch, which is instead preserved in *CCDC113-KO* and *CCDC96-coDel* mutants, is composed of Fap251, whereas the proximal part of the arch, which connects to the N-DRC (NDRC4; Fig 5E, 5F, 5I, and 5J), is likely composed of Ccdc96 and/or Ccdc113. Alternatively, the fragment(s) of Ccdc96/Ccdc113 stabilize the docking of as-yet unidentified subunits of the arch. In agreement with this, the classification and averaging analyses of the axonemal units of *Tetrahymena FAP251-KO* mutants [38] showed that the entire arch is lost in only 49% of the analyzed axonemal repeats, while in the remaining 51% only part of this structure is missing. Accordingly, Fap251 is present in the ciliomes of *CCDC113-KO* and *CCDC96-coDel* mutants (Tables 1, S2 and S3). Thus, Fap251, Ccdc113, and Ccdc96 are likely to be positioned in close proximity to each other but Fap251 docking to the axoneme is Ccdc96 and Ccdc113-independent.

**Table 1:** Selected data obtained during mass-spectrometry analyses of the proteome of cilia isolated from wild-type and mutant cells.

Protein name	Number in TGD	WT	CCDC113-KO	WT	CCDC96-coDel	WT	DRC3-coDel
**Ccdc113**	TTHERM_00312810	33/8	**0**	18/7	**0**	10/7	7/4
			**0**	13/10	**0**	13/10	5/4
**Ccdc96**	TTHERM_00529650	39/16	**3/2**	31/16	**0**	21/15	15/9
			**0**	21/12	**0**	21/12	8/7
**Fap57A**	TTHERM_00105300	14/7	15/9	13/9	22/14	7/6	13/10
			10/7	15/12	10/9	15/12	14/13
**Actin**	TTHERM_00190950	186/16	148/16	96/16	91/14	66/14	78/11
			86/15	65/16	59/18	65/16	54/14
**Drc1**	TTHERM_01345750	37/17	42/23 (1)	38/20	39/17	16/10	18/10
			24/18 (1)	25/15	18/12	25/15	10/9
**Drc2**	TTHERM_00971830	26/11	30/19	20/11	19/11	20/6	14/8
			18/13	8/7	10/8	8/7	2/2
**Drc3**	TTHERM_00316370	48/11	52/21	31/15	34/17	22/12	**0**
			32/16	21/12	17/10	21/12	**0**
**Drc4a**	TTHERM_00857910	30/15	39/17	32/15	46/17	29/10	14/10
			22/16	28/18	21/15	18/18	6/6
**Drc4b**	TTHERM_00649240	37/13	48/21	26/14	42/14	17/8	22/10
			26/15	20/11	11/7	20/11	5/4
**Drc7**	TTHERM_00473320	55/15	48/23	40/22	61/24	22/11	17/9
			37/20	27/18	31/18	27/18	13/12
**RSP3B**	TTHERM_00566810	58/18	81/26	32/12	71/18	36/12	20/8
			37/16	37/17	28/16	36/17	9/6
**PF6**	TTHERM_00430030	132/50	163/56	73/34	92/38	56/28	33/23
			88/41	51/32	44/26	51/32	37/26
**Cfap251**	TTHERM_01262850	39/14	25/13	36/16	56/26	32/17	15/9
			27/15	32/21	29/19	32/21	17/11
**Cfap299**	TTHERM_00427460	22/7	2/2	13/5	0	4/3	4/2
			0	6/4	0	6/4	3/3

Table shows the numbers (X/Y) of all the identified peptides (X, in a Mascot program, all significant matches) and unique peptide sequences (Y, in a Mascot program, significant sequences). Data in two rows represent data from two independent experiments (complete data are presented in S2–S4 Tables). Note that Ccdc113 is missing in *CCDC96-coDel* mutants and Ccdc96 is missing in cilia isolated from *CCDC113-KO* cells and that both Ccdc96 and Ccdc113 are present in cilia of *DRC3-coDel* mutant. One of the control samples (WT) was common for *CCDC96-coDel* and *DCR3-coDel* mutants. The ortholog of *Chlamydomonas* FBB9 (flagellar/basal body protein 9) and mammalian Cfap299 was also reduced or missing in *CCDC113-KO* and *CCDC96-coDel* mutants but this protein was not identified among ciliary proteins co-immunoprecipitating with Ccdc113 or biotinylated in cells expressing Ccdc96-HA-BirA* or Ccdc113-HA-BirA* (see S5–S7 Tables). Radial spoke protein Rsp3B and central apparatus protein Pf6 data are shown to compare the amount of proteins in different samples. TGD–Tetrahymena Genome Database.

We observed that the Ccdc96/Ccdc113 complex forms in total twelve connections to the neighboring structures in wild-type cilia: six with the protofilaments of the A-tubule (MT1-MT6; Fig 5), four with the N-DRC (NDRC1–NDRC4; Fig 5), two with IDA g (IDAg1–IDAg2; Fig 5), and one connection to the base of RS3 (RS3; Fig 5). The absence of the Ccdc96/Ccdc113 complex in the axonemes of the two investigated mutants uncovers a residual small density that, supported by protofilament A5 (MT1; Fig 5), bridges the N-DRC and the base of IDA g (NDRC2 and IDAg1, respectively; Fig 5). The identity of this residual density is not yet known: it could simply be formed by one of the N-DRC proteins or by an as-yet unidentified protein that would directly interacts with Ccdc96/Ccdc113.

The structure and position of the Ccdc96/Ccdc113 complex indicated that it may play a role in the transmission of signals that originated at the central apparatus and through the RS3 are passed to the dynein g and/or the N-DRC. Thus, we propose the Ccdc96/Ccdc113 complex as a key component for the local coordination of the activity of these three ciliary structures.

In *Chlamydomonas* the N-DRC is connected directly to the head of IDA g [17], and such a connection is formed by DRC3 protein [23]. To investigate if ciliary defects caused by the loss of Ccdc96/Ccdc113 and Drc3 are similar, we engineered a *Tetrahymena DRC3-coDel* mutant (S5J Fig). Mass-spectrometry analyses of the protein composition of cilia of *DRC3-coDel* cells revealed that both Ccdc96 and Ccdc113 proteins and N-DRC subunits other than Drc3 (TTHERM_00316370) were present in mutant cilia (Tables 1 and S4). The swimming behavior (Fig 3H) and ciliary beat in *DRC3-coDel* mutants were altered and the waveform and amplitude often varied during subsequent cycles of the analyzed cilium (Figs 3I–3L and S6 and S4 Video). Although the pattern of cilium beating of the *DRC3-coDel* mutant was often similar to that of *CCDC113-KO* and *CCDC96-coDel* mutants (Fig 3I, 3K and S6), the *DRC3-coDel* swimming paths were short but straight (Fig 3H) in contrast to the wavy trajectories of *CCDC96* and *CCDC113* mutants. Thus, likely both the Ccdc96/Ccdc113 complex and Drc3 contribute to the regulation of dynein g activity and consequently cilia beating in similar but not identical ways.

### Axonemal assembly and functional activity of Ccdc96 and Ccdc113 is co-dependent

To verify if Ccdc96 and Ccdc113 are subunits of the same complex, first we searched for potential partner proteins using co-immunoprecipitation and BioID assays. A Ccdc113-HA-BirA* fusion protein expressed under the control of its native promoter localized in cilia (S4H Fig) and rescued the motility of *CCDC113-KO* mutants, although less efficiently than Ccdc113-3HA (WT 337+/-4 μm/sec [SE] n = 76, CCDC113-KO 201+/-4 μm/sec, n = 82, rescued with Ccdc113-BirA* 257 +/– 3 μm/sec [~76% of the wild-type cells], n = 105). Mass-spectrometry analyses of the biotinylated ciliary proteins purified either from cells expressing Ccdc113-HA-BirA* or wild-type cells (control) (S4I–S4K Fig) led to the identification of proteins that are likely positioned in close proximity to Ccdc113 (Tables 2 and S5). These are i) Ccdc96, also biotinylated in cilia of cells expressing Drc1-HA-BirA*; ii) two orthologs of *Chlamydomonas* FAP57, named Fap57A and Fap57C; iii) the subunits of the N-DRC; iv) Act1 (actin); and v) Fap251. Ccdc96 and actin were also biotinylated (Tables 2 and S5) when BirA*-HA tag was attached to the N-terminal end of Ccdc113 (S4M and S4N Fig).

**Table 2:** Biotinylated proteins in cilia of cells expressing BirA*-tagged Ccdc113 or Ccdc96 identified using mass spectrometry.

Protein name	Number in TGD	Ccdc113-BirA*		BirA* Ccdc113	Ccdc96-BirA*		BirA* Ccdc96
		Exp 1	Exp 2	Exp 1	Exp 1	Exp 2	Exp 1
**Ccdc113**	TT_00312810	41/16	25/14	7/7	83/22	41/16	0/0
**Ccdc96**	TT_00529650	2/2	3/3	9/9	77/23	31/17	4/4
**Fap57A**	TT_00105300	45/25	67/34	0	177/47	52/32	0
**Fap57B**	TT_00052490	0	0	0	0	0	0
**Fap57C**	TT_00214710	6/6	1/1	0	36/16	6/5	0
**actin**	TT_00190950	5/3	3/2	2/2	14/7	6/5	2/2
**Drc1**	TT_01345750	1/1	2/1	0	32/16	6/5	0
**Drc2**	TT_00971830	0	0	0	31/10	0	0
**Drc3**	TT_00316370	0	0	0	24/10	8/6	0
**Drc4a**	TT_00857910	1/1	0	0	29/10	6/4	0
**Drc4b**	TT_00649240	0	0	1/1	21/9	7/5	0
**Fap251**	TT_ 01262850	0	2/2	0	0	2/2	0

Table shows the numbers (X/Y) of all the identified peptides (X) and unique peptide sequences (Y). TGD–Tetrahymena Genome Database. Complete data are presented in S5 and S6 Tables. In the case of BirA*-HA-Ccdc96 experiment, actin was also identified in a control sample (2/2; S6 Table). Note that in Ccdc96-HA-BirA* expressing cells biotinylated proteins were identified by a significantly higher number of peptides compared to cells expressing Ccdc113-HA-BirA*. This could be due to: (i) the different distance between BirA* and neighboring proteins; (ii) a more or less exposed position of BirA* (the protein end can be hidden between other proteins); (iii) the conformation of the protein end to which the BirA* ligase is fused; we do not know yet where N- and C-termini of Ccdc96 and Ccdc113 are positioned; (iv) the level of the assortment of the transgene enabling expression of the BirA* tagged protein.

Mass-spectrometry analyses of the biotinylated ciliary proteins isolated from cells expressing Ccdc96-HA-BirA* under the control of its promoter (S5K Fig) confirmed that Ccdc96 protein is positioned in close proximity to Ccdc113, Fap57 orthologs, actin and N-DRC subunits (Tables 2 and S6). When BirA* ligase was attached to the N-terminal end of Ccdc96, mass spectrometry failed to identify proteins other than Ccdc96 and, surprisingly, Dyh7, the motor protein of IDAf/I1 (S4N Fig and S6 Table), suggesting that a weakly conserved fragment of Ccdc96 could extend towards this two-headed dynein arm.

The putative interactions between Ccdc96 and Ccdc113 were further verified by a pull-down assay. GFP-Ccdc96 or GFP (control) were overexpressed in *Tetrahymena* cells and purified from the cytoplasmic fraction using anti-GFP conjugated resin. After incubation with the cytosolic fraction obtained from cells overexpressing Ccdc113-HA, the absorbed proteins were analyzed by Western blot. Only the sample containing GFP-Ccdc96 pulls down Ccdc113-HA protein (S8A and S8A’ Fig). To summarize, Ccdc113 and Ccdc96 proteins are positioned in close proximity and likely form a novel ciliary complex.

Actin and Ccdc96 are the only proteins identified by high peptide number among proteins that also co-immunoprecipitate with Ccdc113-3HA expressed at the native level (S7 Table and S4L Fig), suggesting direct or indirect interactions. Actin is a component of all single-headed IDAs [39] and *Tetrahymena ACT1-KO* cells swim extremely slowly [40]. The presence of actin indicates that Ccdc113 is likely located near one or more single-headed IDAs (which is in agreement with cryo-ET showing a connection between dynein g and a structure composed of Ccdc96/Ccdc113).

Fap251 was detected by the low number of peptides among proteins that are biotinylated in cilia of cells expressing Ccdc113-HA-BirA* (exp. 2; S5 Table) or Ccdc96-HA-BirA* (exp 2; S6 Table), but not among proteins that co-immunoprecipitated with Ccdc113-3HA (S7 Table). Thus, the biochemical data support the ultrastructural analysis showing that the Ccdc96/Ccdc113 complex and part of the arch composed of Fap251 are positioned in close proximity but do not interact (or interact weakly) with each other.

*Tetrahymena* Fap57 orthologs A, B, and C are also biotinylated in cells expressing BirA*-tagged subunits of the tether complex Fap43 and Fap44 [41]. The *Chlamydomonas fap57* mutant lacks an elongated complex connecting different structures in the axonemal unit [42]. Because Fap57A, B and C proteins were identified near T/TH and Ccdc96/Ccdc113 complexes, we speculate that FAP57 orthologs can form or be a part of the elongated structure connecting all major complexes also in *Tetrahymena* cilia.

**Fig 6:**
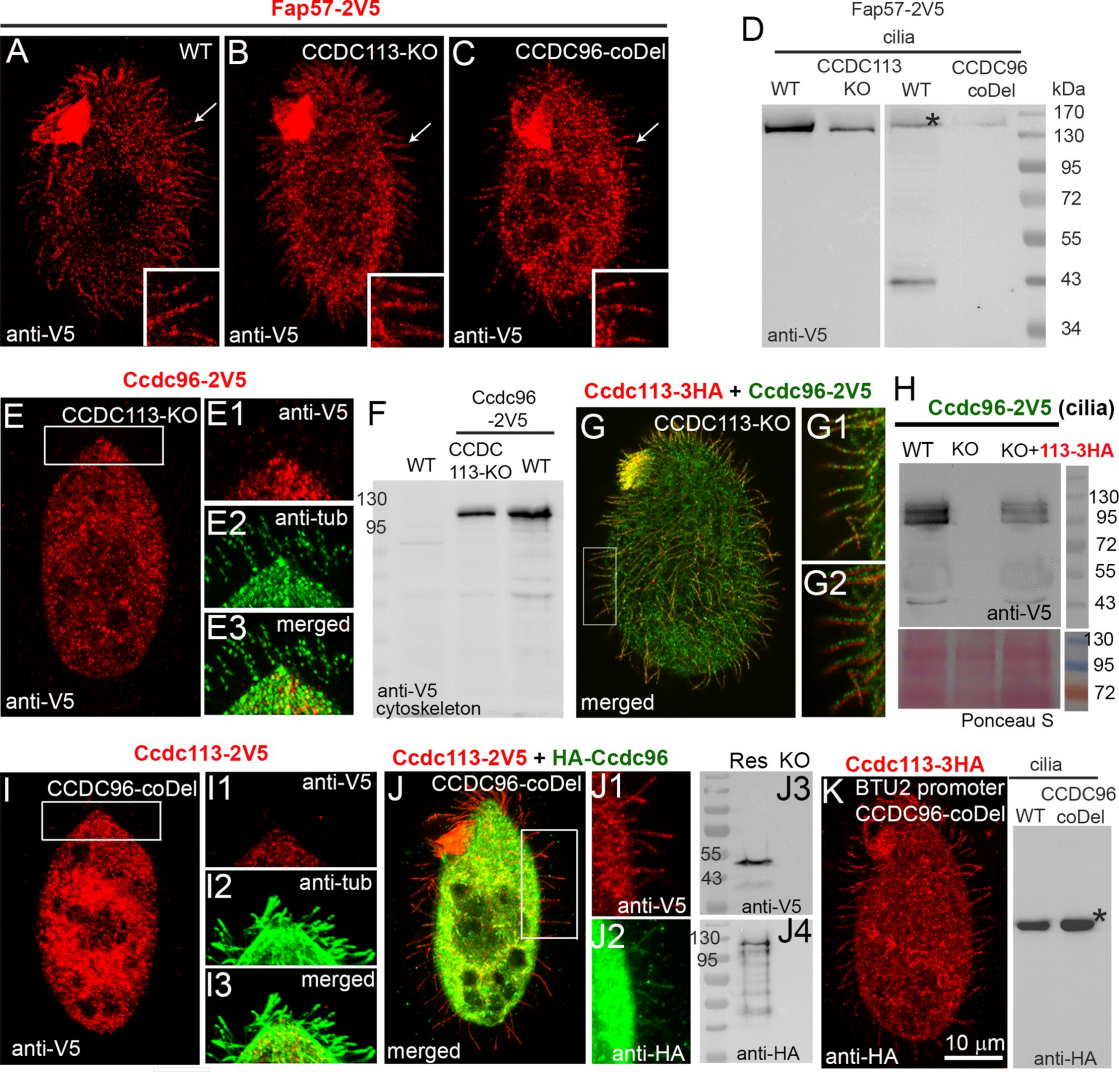
Ciliary localization of Ccdc96 requires the presence of Ccdc113. (A-C) Immunofluorescence confocal images of (A) wild-type (WT), (B) *CCDC113-KO* and (C) *CCDC96- coDel Tetrahymena* cells expressing Fap57A-2V5 at native levels, labeled with anti-V5 antibodies showing that Fap57A localizes in cilia independently of Ccdc113 and Ccdc96. (D) Western blot of ciliary proteins isolated from WT and knockout cells expressing Fap57A-2V5 under the control of its native promoter. Star indicates the position of the Fap57A-2V5 protein. An additional band in one of the WT samples is a partly degraded Fap57A-2V5. In the case of a blot to the right, only 25 μg of proteins were loaded. (E-E3, F) Immunofluorescence confocal image (E) and Western blot (F) showing that Ccdc96-2V5 is not targeted to cilia in cells lacking Ccdc113 (E, E1) although it is present in cells (F). (G-G2) Immunofluorescence confocal images and Western blot (H) indicating that co-expression of Ccdc113-3HA with Ccdc96-2V5 restores ciliary localization of both proteins in *CCDC113-KO* mutants. (I-I3) Immunofluorescence confocal image showing that Ccdc113-2V5 is not targeted to cilia in cells lacking Ccdc96. (J-J4) Immunofluorescence confocal images (J-J2) and Western blot (J3-J4) indicating that co-expression of Ccdc113-2V5 with HA-Ccdc96 restores ciliary localization of both proteins in *CCDC96-coDel* mutants. (K) Immunofluorescence confocal image and Western blot showing that Ccdc113-3HA expressed at higher levels (under the control of the *BTU2* promoter) is present in cilia in cells lacking Ccdc96 (*CCDC96-coDel* mutant).

To investigate if Ccdc113 and Ccdc96 are required for ciliary localization of Fap57A, wild-type, *CCDC113-KO*, and *CCDC96-coDel* mutant cells were transformed with a construct enabling expression of 2V5-tagged Fap57A under the control of its native promoter. Immunofluorescence analysis showed that Fap57A-2V5 is targeted to cilia in both *CCDC113* and *CCDC96* knockout mutants as in wild-type cells (Fig 6A–6D). Moreover, Fap57A and Fap57C were found in the total ciliary proteome of both mutants, and the number of identified peptides was similar to the number of peptides in the proteome prepared from wild-type axoneme (Tables 1, S2 and S3). Thus, Fap57A protein and likely Fap57C do not require either Ccdc96 or Ccdc113 for their ciliary localization.

In contrast, Ccdc96-2V5 expressed under the control of its native promoter was undetectable in cilia of *CCDC113-KO* cells but was present in the cell body (Fig 6E and 6F). Mass-spectrometry analyses of the total cilia proteome confirmed that Ccdc96 was either missing or only present at a very low level in cilia isolated from *CCDC113-KO* cells (Tables 1 and S2). Expression of Ccdc113-3HA from the non-essential *BTU2* locus in *CCDC113-KO* cells carrying the Ccdc96-2V5 transgene restored ciliary localization of both Ccdc96-2V5 and Ccdc113-3HA (Fig 6G and 6H), and led to restored cell motility. Similarly, a Ccdc113-2V5 fusion protein expressed under the control of the native promoter was not targeted to cilia in *CCDC96-coDel* cells (Fig 6I–6I3 and blot to the right, middle row; S8C Fig). Moreover, we observed some degradation of Ccdc113-2V5 (arrowhead; S8B Fig and blot to the left; S8C Fig), suggesting that Ccdc96 could stabilize Ccdc113 within the cell body. Accordingly, Ccdc113 was not found in the *CCDC96-coDel* ciliome (Tables 1 and S3). Expression of HA-Ccdc96 from the *BTU1* locus restored ciliary localization of both fusion proteins, HA-Ccdc96 and Ccdc113-2V5 (Fig 6J–6J4 and blot to the right, first row; S8C Fig) and cell motility. Interestingly, expression of an HA-Ccdc96 A370-Y794 fragment but not an N-terminal Ccdc96 truncation was sufficient to restore ciliary localization of Ccdc113-2V5 in *CCDC96-coDel* mutant (S8D Fig). These data are in agreement with the cryo-ET analyses showing that both Ccdc96 and Ccdc113 are missing in cells with a single gene knockout.

Surprisingly, when Ccdc113-3HA was overexpressed in *CCDC96-coDel* mutants, it localized in cilia (Fig 6K), but the mutants retained slow motility. Similarly, overexpressed HA-tagged Ccdc96 was present in cilia assembled by *CCDC113-KO* mutants. To determine whether overexpressed proteins present in cilia are stably incorporated into the axonemal structure, cells were permeabilized with 1% Triton-X-100 and then fixed with 2% PFA instead of simultaneous fixation and permeabilization [43]. Under such conditions, the ciliary HA-positive signal was diminished in mutants (knockouts of the partner protein). In otherwise wild-type cells expressing the respective tagged protein under the control of the native promoter that were fixed side-by-side, the HA-positive signal was more prominent (S9A–S9B” Fig). The Western blot analyses of the ciliary fractions [44] showed that some of the overexpressed proteins remain bound to the axonemes (S9C–S9G Fig). Thus, in the absence of a partner protein, the other subunit of the Ccdc96/Ccdc113 complex can be effectively transported to cilia, probably docked (S9H Fig) but alone is insufficient to restore cilia motility.

Taken together, our results suggest that (i) Ccdc113 is unstable in *CCDC96-coDel* mutants (S8B and S8C Fig), but a part of the Ccdc113-3HA “escapes” degradation if Ccdc113 is overexpressed. Thus, under native conditions, either Ccdc96 stabilizes Ccdc113 in the cell body or their expression is co-regulated. (ii) When overexpressed, Ccdc113-3HA protein is present within the cell body in *CCDC96-coDel* mutants and can be transported to cilia. Thus, (iii) loading of Ccdc113 onto IFT particles and its transport to the cilium is independent of Ccdc96 (and likewise, transport of Ccdc96 to cilia is independent of Ccdc113). Based on genetic, microscopic, and mass-spectrometry studies, we therefore conclude that Ccdc96 and Ccdc113 are most likely transported to cilia independently but their functional activity in cilia requires the presence of both proteins.

## Conclusions

Using genetic, biochemical, and cryo-ET analyses, we show that Ccdc113 and Ccdc96 jointly form a structure that bridges the base of RS3 with the tail of IDA g and several parts of the N-DRC and is indispensable for proper cilia beating. Moreover, at the base of RS3, Ccdc96/Ccdc113 complex likely partly contributes to the formation of the arch-like structure known to contain Fap251. Since radial spokes are thought to transmit signals that come from the central pair towards the microtubule doublet [26], our data suggest that some of these signals are transmitted by RS3 and “received” by a Ccdc96/Ccdc113 complex to be then transferred to either N-DRC (and possibly other complexes) or dynein g (independently or via N-DRC). The additional connection between N-DRC and dynein g preserved in both *CCDC96-coDel* and *CCDC113-KO* mutants indicates the existence of more than one link between the axonemal structures and highlights the complexity of the mechanisms that regulate ciliary beating. Intriguingly, Ccdc96/Ccdc113 and N-DRC have at least four contact points. The existence of so many connections between these two complexes further points to the complexity of the system regulating cilia beating. In the future, identification of the amino acid residues that form these links would allow elimination of single connections and thus reveal their specific role in the regulation of cilia beating.

Our evidence also further supports the hypothesis that particular IDAs may contribute differently to the regulation of the ciliary waveform and amplitude, as lack of the Ccdc96/Ccdc113 complex affects *Tetrahymena* cilia beating in a different way to that caused by the absence of the tether/tetherhead complex regulating IDAf/I1 or deletion of Dyh6 or Dyh 7, the IDAf/I1 motor domains, [41], or several IDAs [45].

Finally, a growing body of evidence shows that some proteins believed to be exclusively components of motile cilia also play a role in other structures such as the centrosome [46,47]. Consistent with this, our data show that Ccdc96 and Ccdc113, which were originally identified in cells assembling primary cilia as centrosome-associated proteins [31], are structural components of the motile cilia axoneme. In humans, mutations in genes encoding such proteins may lead not only to primary ciliary dyskinesia caused by motile cilia defects but also to more complex multi-symptom disorders.

## Materials and methods

### Cells culture and phenotypic analysis

Wild-type CU428.2 and B2086.2 cells were obtained from the *Tetrahymena* Stock Center (Cornell University, Ithaca, NY, US). Cells of all types were grown in SPP medium [48]. The cell-proliferation and phagocytosis rate were estimated as described previously [38,41]. To measure the length of swimming paths, cells were diluted to a density of 7500 cells/ml and a drop of cells was placed on the bottom of the Petri dish. Swimming cells were recorded at room temperature using a Zeiss Discovery V8 Stereo microscope (Zeiss, Germany) equipped with Zeiss Plans 10× FWD 81 mm objective and Axiocam 506 camera and ZEN2 (blue edition) software. The length of the paths was measured using ImageJ software. Colored lines parallel to the paths were drawn on a new layer using Adobe Photoshop CC software and slightly shifted to show the original paths.

To analyze cilia beating, a 15 μl drop of cells in a culture medium mixed 1:10 with 20% Ficoll in 10 mM Tris-HCl, pH 7.5 was placed on the glass slide between two strips of adhesive tape, covered with a coverslip, and cilia beating was recorded in at least 10 cells using a high-speed camera (Andor Zyla 5.5 sCMOS) mounted on a Leica DMI 6000 microscope (63x oil immersion lens, numerical aperture 1.4) with an Andor DsD2 unit. Video was recorded at 200 frames/s. To establish the position of the cilium during the power and recovery stroke, the subsequent frames were analyzed, and based on these frames, the position of the cilium was determined. The power and recovery stroke were established based on the direction and plane of the cilia movement (perpendicular versus parallel to the cell surface). To estimate the beat frequency, videos in which cilia could be clearly observed were selected and the number of frames showing complete full beat cycle(s) was scored.

To calculate the cilia beat amplitude, high-speed videos were recorded as “.tif” images and cilium position was marked in successive frames using ImageJ and Adobe Photoshop image editors. To measure the amplitude of the cilium at 1.5 μm above the cell surface, lines parallel to the cell surface were marked at the level of cell surface and 1.5 μm above. The ciliary amplitude was expressed as the angle with vertex at the point of the cilium base at the lower measuring line and arms crossing the points where the most external cilium traces intercepted the upper measuring line [49].

### Genome modifications

The transgenes enabling expression of the tagged protein in its native locus, protein overexpression, or gene knockout were prepared using Phusion Hot Start II high-fidelity DNA polymerase (Thermo Fisher Scientific Baltics, Lithuania) and genomic DNA purified from CU428.2 strain as a template. All primers are listed in S8 Table. The transgene enabling germ-line knock-out of *CCDC113* was constructed using the pNeo4 plasmid [50] and *CCDC113* heterokaryons were generated as described previously [51,52]. Mutant strains with deleted *CCDC96* or *DRC3* were engineered using targeted ectopic DNA elimination in macronuclei (co-Deletion) and the pMcoDel plasmid, which was kindly provided by Dr. K. Mochizuki [37]. To express analyzed proteins under the control of the native promoter in the native locus, fragments of an open reading frame and 3’UTR were removed from pFAP44-3HA, pFAP44-2V5, or pFAP44-HA-BirA* plasmids [41] using MluI and BamHI in the case of the open reading frame and PstI and XhoI in the case of the 3’UTR. The fragments were replaced with fragments of the open reading frame and 3’UTR of the analyzed gene amplified by PCR (see S8 Table). To express Ccdc96 or Ccdc113 proteins with an N-terminal BirA*-HA tag in a native locus, we modified the *MTT1-GFP* overexpression plasmid by replacing the GFP coding region with the BirA*-HA coding region and 5’UTR of *BTU1* by 5’UTR of the analyzed gene and inserting a neo2 cassette to enable selection of the positive transformants, and strengthen the phenotypic assortment.

To obtain a transgene enabling protein tagging and overexpression, the open reading frame of the analyzed gene was amplified by PCR and cloned into an *MTT1*-*HA* [53] or an *MTT1-GFP* [54] plasmid. Protein expression was controlled by the cadmium-inducible *MTT1* promoter [55].

For rescue experiments, *CCDC113-KO* mutant cells were transformed with a transgene enabling expression of a C-terminally 3HA-tagged Ccdc113 under the control of the *BTU2* promoter in the *BTU2* locus. A transgene was constructed by insertion of the *BTU2* promoter just before the *CCDC113* open reading frame into a plasmid enabling expression of Ccdc113-3HA from its native locus. Next, the plasmid was digested with EcoRV and ApaI to remove the Neo4 cassette and the 3’UTR of *CCDC113*, and the fragment was replaced with a pPur4 cassette [56] followed by the 3’UTR of *BTU2*. To rescue *CCDC113-KO* cells with a transgene enabling expression of Ccdc113-HA-BirA*, the 3HA coding region in the CCDC113-3HA overexpression plasmid was replaced with the HA-BirA* coding region [41].

To rescue *CCDC96-coDel* mutants, cells were transformed with a ~3 kb fragment amplified by PCR containing the 5’UTR (without ATG) and a coding region (with ATG) of CCDC96 separated by a coding region of the HA tag. Rescued cells were selected based on the restored motility.

After separation from the plasmid backbone (uncut plasmid in the case of the co-Deletion construct), DNA was precipitated onto DNAdel Gold Carrier Particles (Seashell Technology, La Jolla, CA) and *Tetrahymena* cells were transformed using the Biolistic PDS-1000/He Particle Delivery System (Bio-Rad, US). The transgenes were introduced into macro- or micronuclear genome via homologous recombination [36,51,52]. Transformed cells were selected using an appropriate drug and assorted as described previously [36,41].

### Immunofluorescence and biochemical analyses

Immunofluorescence, Western blot, and proximity labeling assay were performed as described previously [38,57]. For immunofluorescence, cells were fixed on coverslips with a mixture of 1% Triton-X-100 and 2% PFA, and after drying and blocking with 3% BSA/PBS, incubated overnight at 4 ^o^C with primary antibodies diluted in 3% BSA/PBS (130 mM NaCl, 2 mM KCl, 8 mM Na_2_HPO_4_, 2 mM KH_2_PO_4_, 10 mM EGTA, 2 mM MgCl_2_, pH 7.2) at the following concentrations: monoclonal mouse anti-HA.11 (cat. 901503, BioLegend, San Diego, CA) 1:300, polyclonal rabbit anti-HA (C29F4, Cell Signaling Technology, Leide, The Netherlands) 1:300, monoclonal rabbit anti-V5 (D3H8Q, Cell Signaling Technology, Leide, The Netherlands) 1:1000, anti-centrin 20H5 (cat. 04–1624, Merck Millipore, Billerica, MA) 1:300, and anti-α-tubulin 12G10 (Developmental Studies Hybridoma Bank, Iowa University, Iowa City, IA) 1:100. After washing 3 x 5 min in PBS, coverslips were incubated for 1.5 hours at room temperature with the appropriate secondary antibodies diluted in 3% BSA/PBS: anti-mouse-IgG-Alexa Fluor 488, anti-mouse-IgG-Alexa Fluor 555, anti-rabbit-IgG-Alexa Fluor 488, anti-rabbit-IgG-Alexa Fluor 555, all in dilution 1:300 (Invitrogen, Life Technologies, Eugene, OR). Coverslips were mounted in Fluoromount-G (Southern Biotech, Birmingham, AL) and cells were viewed using a Zeiss LSM780 or a Leica TCS SP8 confocal microscope using a 63x Plan Apochromat oil immersion lens, numerical aperture 1.4, and Zeiss Zen 2012 Blue Edition software.

For Western blot (SDS-PAGE) analyses, if otherwise not indicated, we loaded 30 μg of ciliary or cytoskeletal proteins. For total protein analysis, a total extract from 10^5^ cells (protein overexpression) or 2x10^5^ cells (expression under a native promoter) was loaded. For 2-dimensional analysis, cilia were isolated from cells expressing Ccdc113-3HA under the control of the native promoter and de-membraned, and then axonemal proteins were precipitated using a 2D Cleanup kit (Bio-Rad, Hercules, CA). Approximately 30 μg of the axonemal proteins was loaded onto 7 cm ReadyStrip pH 7–10 strips (Bio-Rad, US) and separated in the PROTEAN IEF instrument (Bio-Rad, US) followed by SDS-PAGE and Western blot analysis. The primary antibodies were diluted in 5% milk/TBST (10 mM Tris-HCl, 150 mM NaCl, 0.5% Tween20, pH 7.5) as follows: monoclonal mouse anti-HA (1:3000), monoclonal rabbit anti-V5 (1:1000), and monoclonal mouse anti-α-tubulin 12G10 (1:10 000).

For the BioID assay, we engineered *Tetrahymena* cells expressing one of the following fusion proteins: Ccdc96-HA-BirA*, Ccdc113-HA-BirA* or Drc1-HA-BirA* under the control of the respective native promoter or BirA*-HA-Ccdc96 and BirA*-HA-Ccdc113 under the control of the *MTT1* promoter (cells were grown without cadmium to avoid protein overexpression). Cells at a density of 2 × 10^5^ cells/ml were starved overnight in 10 mM Tris–HCl buffer, pH 7.5 and incubated in the same buffer supplied with 50 μM biotin for 4 h at 30°C. After deciliation [58], cilia were resuspended in 0.5 ml of axoneme stabilization buffer (20 mM potassium acetate, 5 mM MgSO_4_, 20 mM HEPES, pH 7.5, 0.5 mM EDTA with protease inhibitors (Complete Ultra EDTA-free; Roche, Indianapolis, IN)) and incubated for 5 min on ice with addition of 0.2% NP-40 to remove the ciliary membrane. After spinning down, the collected axonemes (10 min at 21,100×*g* at 4°C) were lysed for 1 hour (0.4% SDS, 50 mM Tris–HCl, pH 7.4, 500 mM NaCl, 1 mM DTT with protease inhibitors) at RT, centrifuged (8000×*g* at 4°C) and the obtained supernatant was diluted with three volumes of 50 mM Tris–HCl buffer, pH 7.4 and incubated overnight with 100 μl of streptavidin-coupled Dynabeads (Dynabeads M-280 Streptavidin, Thermo Fisher Scientific, Waltham, MA, USA) at 4°C. After washing (6 × 5 min with washing buffer: 15 mM Tris–HCl, pH 7.4, 150 mM NaCl, 0.1% SDS, 0.3 mM DTT) at 4°C, the biotinylated, resin-bound proteins were analyzed by mass spectrometry (Laboratory of Mass Spectrometry, Institute of Biochemistry and Biophysics, PAS, Warsaw, Poland) and by Western blot with Pierce High Sensitivity-streptavidin-HRP (Thermo Scientific, Rockford, IL) diluted 1:40,000 in 3% BSA/TBST. The proteins detected by the high number of peptides are likely to be positioned in close proximity to the BirA*-tagged protein. Note that because the entire biotinylated proteins (not biotinylated peptides) were bound to the resin, the large but poorly biotinylated proteins (such as dynein heavy chains) can be detected by the similar number of peptides as small proteins but moderately biotinylated.

For immunoprecipitation, cilia from the *Tetrahymena* cells expressing Ccdc113p-3HA at the native level and wild-type cells (control) were harvested as described [58] and suspended in 10 mM Tris-HCl buffer, pH 7.5, with protease inhibitors (Complete Ultra EDTA-free; Roche, Indianapolis, IN), and combined with an equal volume of 2% NP-40 and 1 M NaCl in 80 mM Tris-HCl buffer, pH 7.5. After 20 min incubation on ice, the isolated axonemes were pelleted at 20,000 × *g* for 20 min and washed with 0.5 M NaCl, 40 mM Tris-HCl, pH 7.5, pelleted again for 30 min and extracted with 0.5 M KI, 30 mM NaCl, 5 mM MgSO_4_, 0.5 M EDTA, 1 mM dithiothreitol, and 10 mM HEPES, pH 7.5, also on ice [44]. After centrifugation at 16,000 × *g* for 15 min at 4°C, the supernatant was diluted 250 x with 10 mM Tris-HCl, pH 7.5 to reduce KI concentration, and proteins were concentrated to 1.8 mg/ml by ultrafiltration in Vivaspin columns (Sartorius, Goettingen, Germany).

A concentrated supernatant was incubated with anti-HA-conjugated resin (Pierce HA Epitope Tag Antibody Agarose conjugated, Thermo Scientific, Rockford, IL) with rotation overnight at 4°C according to the manufacturer’s instructions. Resin was washed six times for 5 min with 1 ml of 10 mM Tris-HCl, pH 7.4, 150 mM NaCl, and 0.5 mM EDTA at 4°C. Proteins were separated on 10% SDS–PAGE gel and silver stained, or were analyzed by mass spectrometry.

For pull-down assays, cells with introduced transgenes enabling overexpression of either Ccdc113-HA or GFP-Ccdc96, or GFP alone (control) were grown for 3 hrs in SPP medium supplemented with 2.5 μg/ml CdCl_2_ to induce protein overexpression, washed with Tris-HCl pH 7.5 buffer, centrifuged, and resuspended in TBS buffer (20 mM Tris-HCl, pH 7.5, 300 mM NaCl with protease inhibitors). Next, cells were disrupted using a French press (French Pressure Cell Press Model FA-078, ThermoSpectronic) and centrifuged at 20,000g for 15 min at 4 ^o^C to remove cell debris. The collected supernatant was ultracentrifuged at 100,000 × *g* for 1 h at 4 ^o^C. Samples of the supernatant collected from GFP or GFP-Ccdc96-expressing cells and containing 1 mg of protein, were incubated for 1 h with anti-GFP-conjugated resin (GFP-Trap-Agasose, ChromoTek, Germany) on a shaker at 4 ^o^C. After washing (5 x 5 min) with TBS buffer (10 mM Tris-HCl, pH 7.5, 150 NaCl, 0.5 mM EDTA with protease inhibitors), resin with bound GFP or GFP-Ccdc96 was incubated with supernatant obtained from Ccdc113-HA-expressing cells (1 mg of proteins) for 1 h at 4 ^o^C with shaking. After washing (as above), precipitated proteins were separated from the resin with a loading buffer and analyzed by Western blot using polyclonal anti-GFP (1:2000, ab6556, Abcam) or monoclonal anti-HA (1:2000) antibodies.

For the entire cilia proteome, cilia were isolated as described [58]. Approximately equal amounts of ciliary proteins (0.2–0.25 mg) from wild-type and mutant cells were run on an SDS-PAGE gel and analyzed by mass spectrometry (Laboratory of Mass Spectrometry, Institute of Biochemistry and Biophysics, PAS, Warsaw, Poland).

### Axoneme isolation for cryo-ET

*Tetrahymena thermophila* axonemes from wild-type cells (strain CU428) and mutants (*CCDC113-KO* and *CCDC96-coDel*) were isolated using the dibucaine method [59]. Briefly, cilia were detached from the cells and purified by centrifugation twice at 2,400 × *g*, 4°C, for 10 min. Purified cilia were demembranated using 1% IGEPAL CA-630 in HMEEK buffer (30 mM HEPES, pH 7.4, 5 mM MgSO_4_, 1 mM EGTA, 0.1 mM EDTA, and 25 mM KCl), and axonemes were collected by centrifugation at 10,000 × *g*, 4°C, for 10 min. The axoneme pellet was carefully resuspended in HMEEK buffer and stored on ice for cryo-sample preparation within 4 hrs.

### Preparation of cryo-TEM grids

Isolated cilia in solution were applied on glow-discharged TEM grids with holey carbon support film (Quantifoil Micro Tools, R 3.5/1). Colloidal gold particles (10 nm) were added to the sample to be used as fiducial markers during tomogram reconstruction. The samples were blotted from the back with Whatman filter paper, rapidly frozen in liquid ethane at −182°C using a Leica EM Grid Plunger, and stored in liquid nitrogen until image acquisition.

### Cryo-electron tomography

Data were acquired with an FEI Titan Halo transmission electron microscope operated at 300 kV equipped with a field emission gun (FEG), a Gatan energy filter using a slit width of 20 eV, and a Gatan K2 direct detector. SerialEM software was used for the automated tomographic tilt series acquisition [60]. Full grid montages at low magnification were acquired to find suitable cilia connected to the cell body. The nominal image magnification was ×30,000, resulting in a calibrated pixel size of 2.36 Å in the super-resolution mode of the camera. Tilt series were recorded with 2° increments with a bidirectional tilt scheme from –20° to 64° and –64° (when possible). The defocus range was –4 to –5 μm, and the cumulative dose was 130–150 e per Å2 per tomogram. Images were acquired in the dose fractionation mode with frame times between 0.10 and 0.25 s.

### Image processing and subtomogram averaging

Frames were aligned using the K2Align program (IMOD v.4.9.3), and tomogram reconstruction was performed using Etomo (IMOD v.4.9.3) [61] with weighted back projection. Contrast transfer function curves were estimated with CTFPLOTTER and corrected by phase-flipping with the software CTFPHASEFLIP, both implemented in IMOD [62]. The tomograms were binned 3 times, resulting in a pixel size of 0.7 nm. For particle picking and visualization of tomograms, a nonlinear anisotropic diffusion filter by IMOD [61] was applied to enhance the contrast of macromolecular structures.

Subtomogram averaging (S9 Table) was performed on the unfiltered tomograms with PEET v.1.11.0 from the IMOD package. Particles were picked exclusively from the round axonemes and from all the doublets in order to minimize the influence of the missing wedge in 96-nm distances. The center of the particles was on the base of radial spoke 3 [63].

Visualization of tomograms and average densities was performed in 3dmod from IMOD, and rendering of isosurfaces was performed using UCSF Chimera [64].

Confidence maps were generated with the false discovery rate control method according to reference [65] in order to validate our averaged maps and the identified connections between several protein complexes of the 96-nm repeat. The resolution of the three final 3D model (wild type = 35 Å, CCDCKO96 = 34 Å, CCDCKO113 = 35 Å) was estimated with Fourier Shell Correlation (FSC) and cutoff at 0.143 (S7 Fig).

The cryo-ET density maps have been deposited in the Electron Microscopy Data Bank under accession nos. EMD-12119 (wild type), EMD-12120 (CCDC113-KO), and EMD-12121(CCDC96-del).

### Phylogenetic analyses

The Ccdc96 and Ccdc113 homologs were obtained from the NCBI protein database using Blastp search and either human or *Tetrahymena* proteins as bait. Protein amino acid sequences were aligned using ClustalX2 software [66] and edited using SeaView [67]. The identical and similar amino acid residues were shaded using GeneDoc [68]. The phylogenetic tree was calculated (www.phylogeny.fr) [69–74] and tree was drawn using iTOL (https://itol.embl.de) [75]. The expression profiles of CCDC96 and CCDC113 were checked in GEO Profiles (https://www.ncbi.nlm.nih.gov/geoprofiles) [76].

## Supporting information

S1 Fig Model of the 96-nm-linker structure. (A) A 96-nm linker (green dotted outlines) bridges structures throughout the whole 96-nm repeat, extending from the tail of IDA g (pink) to the heavy chain β of IDA I1/f (pink), going through the N-DRC (navy blue), MIA-like complex (light green) and IC/LC (purple) of the IDA I1/f. (B) Tomographic slices of 96-nm linker.
(TIF)

S2 Fig Ciliary localization of Drc1-HA-BirA* fusion protein in *Tetrahymena*. (A-C) Immunofluorescence confocal images of *Tetrahymena* cells expressing Drc1-HA-BirA* at the native level, double labeled with anti-HA (A) and anti-tubulin (B) antibodies. (C) Merged image of A and B. (C’) A magnified fragment of (C) indicated by a while inset. Note that red and green channels were slightly shifted to better visualize localization of Drc1-HA-BirA* in cilia. (D) Western blot of the ciliary proteins isolated either from wild-type cells (WT) or cells expressing Drc1-HA-BirA* under the control of a native promoter. A star indicates the position of the Drc1-HA-BirA* protein (~126 kDa). The additional, faster migrating bands are most likely partly degraded Drc1-HA-BirA* fusion protein. (E) Detection of the biotinylated proteins in cilia isolated from either WT cells or cells expressing Drc1-HA-BirA* at native levels grown in medium supplemented with biotin for 4 hrs.
(TIF)

S3 Fig Multiple alignments and phylogenetic trees of Ccdc113 and Ccdc96 homologous sequences. Ccdc96 and Ccdc113 homologs were obtained from the NCBI protein database using Blastp search and either human or *Tetrahymena* proteins as bait. Protein amino acid sequences were aligned using ClustalX2 software [66] and edited using SeaView [67]. The identical and similar amino acid residues were shaded using GeneDoc [68]. The phylogenetic tree was calculated (www.phylogeny.fr) [69–74] and the tree was drawn using iTOL (https://itol.embl.de) [75]. The branch support values are shown as %. The coiled-coil domains (blue bars) were predicted using SMART **(**http://smart.embl-heidelberg.de/) [77] and COILS (https://embnet.vital-it.ch/software/COILS_form.html) [78]. Ccdc113 orthologs used: *Branchiostoma floridae* (Bf, XP_002594168.1), *Chlamydomonas reinhardtii* (Cr, XP_001703742.1), *Ciona intestinalis* (Ci, XP_002125206.1), *Ectocarpus siliculosus* (Ec, CBJ30690.1), *Gonium pectoral* (Gp, KXZ50957.1), *Homo sapiens* (Hs, NP_054876.2), *Microplitis demolitor* (Md, XP_008557297.1), *Orussus abietinus* (Oa, XP_012276405.1), *Paramecium tetraurelia* (Pt, XP_001431423.1), *Phytophthora infestans* (Pi, XP_002997358.1), *Saccoglossus kowalevskii* (Sk, XP_002741623.1), *Strongylocentrotus purpuratus* (Sp, XP_785529.1), *Tetrahymena thermophila* (Tt, XP_001033462.1, TTHERM_00312810), *Volvox carteri f*. *nagariensis* (Vc, XP_002949615.1), *Xenopus tropicalis* (Xt, AAH89076.1). Ccdc96 orthologs used: *Branchiostoma floridae* (Bf, XP_002603613.1), *Chlamydomonas reinhardtii* (Cr, XP_001697427.1), *Ciona intestinalis* (Ci, XP_002126679.1), *Danio rerio* (Dr, NP_001122170.1), *Homo sapiens* (Hs, NP_699207.1), *Paramecium tetraurelia* (Pt, XP_001455440.1), *Pseudocohnilembus persalinus* (Pp, KRX11190.1), *Saccoglossus kowalevskii* (Sk, XP_002733290.1), *Tetrahymena thermophila* (Tt, XP_001032676.1), *Xenopus tropicalis* (Xt, XP_002938310.2).
(DOCX)

S4 Fig Lack of Ccdc113 affects cilia-dependent processes. (A) Two-dimensional analyses of axonemal proteins (30 μg) purified from cells expressing Ccdc113-3HA under the control of the native promoter. Isoelectric focusing was performed using 7 cm 7–10 ready-strips. The theoretical calculated pI = 8.87 (https://web.expasy.org/compute_pi). Note that all isoforms are more acidic, suggesting posttranslational modification. (B, B’) Changes in the *CCDC113* locus in engineered knockout cells. (B) A schematic representation of the *CCDC113* locus in a wild-type (WT) and *CCDC113-KO* cells. Blue rectangles represent the *CCDC113* open reading frame, grey rectangles represent 5’ and 3’ UTRs. A white rectangle marks the position of a neo4 cassette that replaced a fragment of the 5’UTR and the open reading frame. Arrows indicate the annealing positions of the primers used to test alteration in *CCDC113* locus. (B’) PCR analysis of the *CCDC113* locus showing that part of the *CCDC113* gene is deleted. PCR amplification of a fragment of the unrelated *FAP208* locus was performed to verify the quality of isolated genomic DNA. (C-E) Knockout of *CCDC113* does not affect cilia assembly and cilia length. Immunofluorescence confocal images of WT (C) and *CCDC113-KO* cells (D) stained with anti-α-tubulin antibodies. Scale bar = 10 μm. (E) Graphical representation of cilia length measurements of WT (white bar, 6.36 μm +/- 0.61, n = 60) and *CCDC113-KO* (grey bar, 6.7 μm +/- 0.77, n = 60) cells. Bars represent standard deviation. (F-G) Expression of Ccdc113-3HA restores normal phagocytosis and proliferation rates. (F) Graphical representation of the proliferation rate of WT, *CCDC113-KO* and *CCDC113-KO* rescued cells. (G) Graphical representation of the efficiency of the formation of food vacuoles. Cells were grown in medium supplemented with India ink and the number of India ink-filled food vacuoles per cell was scored. (H) Immunofluorescence analyses showing that Ccdc113-HA-BirA* localizes in cilia. (I-K) Detection of the biotinylated proteins: (I) in cilia isolated from either WT cells or cells expressing Ccdc113-HA-BirA* at native levels grown in medium supplemented with biotin for 2, 4 or 6 hours; (J-K) ciliary proteins in Ccdc113-HA-BirA* input, unbound and bead-bound fractions. Note that only one major band of biotinylated protein(s) appears in cilia purified from WT cells. Predicted molecular weights of the BirA* tagged proteins: 78 kDa (Ccdc113), 129 kDa (Ccdc96) and 187 kDa (Fap57A). (L) Silver-stained gel showing proteins immunoprecipitated from a ciliary fraction of cells expressing Ccdc113-3HA at native level using beads coated with anti-HA antibodies. (M) Immunofluorescence analyses showing that BirA*-HA-Ccdc113 localizes in cilia. (N) Detection of the biotinylated proteins in cilia isolated from either wild-type (WT) or cells expressing BirA*-HA-Ccdc113 or BirA*-HA-Ccdc96. Numerical data are in S10 Table.
(TIF)

S5 Fig Ccdc96 is required for normal cilia function. (A-C) Immunofluorescence confocal images of *Tetrahymena* cells overexpressing either HA-Ccdc96 full length protein (A), N-terminal fragment M1-I 431 (B), or C-terminal fragment A370—Y794 (C). Note that the C-terminal domain is indispensable and sufficient for protein ciliary localization. (D) Western blot of the total cell extract obtained from cells overexpressing truncations or full-length Ccdc96 protein. Note that Ccdc96 is prone to degradation in total extract from *Tetrahymena* cells. (E-F) Alteration in the *CCDC96* locus in engineered *CCDC96-coDel* cells. (E) Schematic of the *CCDC96* locus in wild-type (WT) and *CCDC96* mutant cells obtained using the co-Deletion method. Blue rectangles represent the *CCDC96* open reading frame, grey rectangles represent 5’ and 3’ UTRs. Arrows indicate the annealing positions of the primers used to analyze the extent of deletion in the *CCDC96* locus. (F) PCR analysis of the *CCDC96* locus in independently obtained deletion mutants with the indicated primers (see S5E) annealing about 1kb upstream and 1 kb downstream of the gene fragment (targeting DNA) inserted into the pMcoDel plasmid. Note that the PCR fragment amplified using WT genomic DNA as a template (control) is larger than PCR fragments amplified using genomic DNA isolated from mutants, indicating deletion of the fragment of the gene. (G) Lack of Ccdc96 does not affect cilia length. Staining of *CCDC96-coDel* cell with anti-α-tubulin antibodies (12G10) revealed that mutant cells assemble cilia of a similar length (6.2 μm +/- 0.54 (standard deviation), n = 50) to WT cells (6.36 μm +/- 0.61, n = 60). (H) Deletion of *CCDC96* reduces proliferation rate. (I) Graphical representation of the efficiency of the formation of food vacuoles. On average (data from three independent experiments), WT cells formed 5.9 vacuoles (n = 300 cells), *CCDC96-coDel* mutants 4.8 vacuoles (n = 300) and *CCDC96-coDel* rescued cells 6.2 vacuoles (n = 300, not shown). (J) PCR analysis of the *DRC3* locus with primers annealing about 1kb upstream and 1 kb downstream of the gene fragment inserted into pMcoDel plasmid. Note that the PCR fragment amplified using WT genomic DNA as a template is significantly larger than PCR fragments amplified using genomic DNA isolated from mutants, indicating deletion of the fragment of the *DRC3* gene. (K) Detection of the biotinylated proteins in cilia isolated from either WT cells (line to the left) or cells expressing Ccdc96-HA-BirA* at native level: input, unbound and beads bound fractions. Numerical data are in S10 Table.
(TIF)

S6 Fig Cilia of *CCDC113*, *CCDC96*, and *DRC3* knockout cells beat with reduced amplitude. Additional analyses of the ciliary amplitude (schematic representation of all recorded consecutive positions of the cilium during the power and recovery stroke).
(TIF)

S7 Fig Resolution of subtomogram averages. Assessment of structural measurements performed on averaged data of axonemal 96-nm repeats from the wild type (wt) and two types of mutant cells (CCDC113-KO, CCDC96-coDel) of *Tetrahymena thermophila*. Fourier Shell Correlation (FSC) curves of the average electron density map from Tetrahymena 96-nm repeats depicting the resolution associated with typical criteria (FSC  =  0.143).
(TIF)

S8 Fig Ccdc96 and Ccdc113 interact with each other. (A, A’) Cytoplasmic GFP-Ccdc96 but not GFP can pull down Ccdc113-HA. Note that overexpressed GFP-Ccdc96 present in the cytoplasm is prone to degradation (stars mark weakly visible non-degraded and partly degraded GFP-Ccdc96. (A’) A longer exposure of the same blot to better visualize less degraded forms of GFP-Ccdc96. (B, C) Western blot of the cytoskeletal (B and C, left panel) and ciliary (C, right panel) proteins isolated from WT cells and cells expressing Ccdc113-2V5 either in WT or *CCDC96-coDel* background, or in *CCDC96-coDel* mutants rescued with HA-Ccdc96 expression. Note that HA signal is not detected in WT cells (B) and that Ccdc113-3HA is partly degraded (arrow head) in cells lacking Ccdc96 (B and C, left panel). (D) Western blot of the ciliary proteins isolated from cells expressing Ccdc113-2V5 either in WT or *CCDC96-coDel* background, or in *CCDC96-coDel* mutants rescued with HA-tagged N-terminal fragment (M1-I431, named N-HA) or C-terminal fragment (A370-Y794, C-HA) of Ccdc96 protein. Note that Ccdc113-2V5 was detected in WT cells or cells rescued with the C-terminal fragment of Ccdc96 (blot to the left) but not with the N-terminal fragment that is not targeted to cilia (blot to the right).
(TIF)

S9 Fig Ccdc96 and Ccdc113 are transported to cilia and likely docked to the axoneme independently of the partner protein. (A-B”) Comparison of the level of HA signal in cells either expressing HA-tagged Ccdc113 (A-A”) or Ccdc96 (B-B”) under the control of the respective native promoter in otherwise wild-type background (nat) or overexpressing these proteins (oex) in a mutant background (deletion of the partner proteins). Note only a weak HA-positive signal in cilia of knockout cells compared with wild-type cells expressing tagged proteins at the native level. (A, B) polyG (anti-polyglycylation antibody to visualize cilia), (A’, B’) anti-HA antibody, (A”, B”) enlarged fragments of A’ and B’, respectively. (C-C”) Western blot of the total ciliary proteins (lines 1–4) and axonemal pellet remaining after 0.5 M KI treatment (lines 5–8) showing the level of Ccdc113 and Ccdc96 proteins in cilia and amount of the proteins bound to the axoneme. Cilia were isolated from: wild-type cells expressing Ccdc113-3HA under the control of the native promoter (113 nat), *CCDC96-coDel* cells overexpressing Ccdc113-HA (113 oex), wild-type cells expressing Ccdc96-3HA under the control of the native promoter (96 nat), *CCDC113-KO* cells overexpressing Ccdc96-HA (96 oex), (C’) Longer exposure of a blot presented in (C). (C”) The level of tubulin in the analyzed samples (a loading control). (C, C’) Note slightly lower level of the proteins in samples obtained from knockout cells overexpressing partner protein (lines 6 and 8) compared to the wild-type cells expressing the same partner protein (lines 5 and 7) suggesting less effective binding of the protein to the axonemes. (D-G) Western blot analyses of total ciliary proteins (T) and ciliary proteins present in the cilia fractions (supernatant (SN) and pellet (P)) obtained after 1% NP-40 treatment, extraction of the axonemes from NP-40 pellet with the 0.6 M NaCl and extraction of the axonemes collected after NaCl treatment, with 0.5M KI (according [44]). Proteins were visualized using Ponceau S and HA-tagged proteins were detected using anti-HA antibodies. (H) A scheme of the proposed model. In wild-type cells, both Ccdc96 and Ccdc113 are stable in the cell body, transported to cilia and docked to the axoneme. In *CCDC96-coDel* and *CCDC113-KO* mutants, a partner protein is degraded within the cell body and thus not transported to cilia. When partner proteins are overexpressed in the mutant background, some of them “escape” degradation and can be transported to cilia and docked to the axoneme, although it remains unknown (?) if the strength of the binding is the same as in wild-type cells where both proteins are present.
(TIF)

S1 Table Mass-spectrometry analysis of proteins biotinylated in cells expressing Drc1-HA-BirA*. Proteins present in both control and experimental samples are highlighted in green.
(XLSX)

S2 Table Comparison of the protein composition of cilia isolated from wild-type (WT) and *CCDC113-KO* cells using mass spectrometry. About 0.2 mg of ciliary proteins was analyzed in WT and *CCDC113-KO*, sample 1, and about 0.1 mg of proteins in the case of *CCDC113-KO*, sample 2. Proteins present in both control and experimental samples are highlighted in green (exp1) or blue (exp2). In previously published cilia and flagella proteomes [32,79–81] the number of the identified proteins was e.g. 1134 proteins (482 identified by 1 peptide) in *Chlamydomonas* flagella [32] or 751 proteins in *Trypanosoma* flagella [81]. A higher number of the identified ciliary protein candidates in *Tetrahymena* ciliome could be due to: (i) differences in the cilia preparation method and analysis of whole cilia versus ciliary fractions, (ii) initial amount of ciliary proteins that were analyzed by mass spectrometry, (iii) presence of more than one ortholog of some ciliary proteins (e.g ciliary dyneins) in *Tetrahymena*; (iv) presence of ciliate-specific proteins as we determined by BlastP search using NCBI database. A similar number of putative ciliary proteins was obtained in our previous studies [41].
(XLSX)

S3 Table Comparison of the protein composition of cilia isolated from wild-type and *CCDC96-coDel* cells using mass spectrometry. About 0.2 mg of ciliary proteins was analyzed in each sample. Proteins present in both control and experimental samples are highlighted in green.
(XLSX)

S4 Table Comparison of the protein composition of cilia isolated from wild-type and *DRC3-coDel* cells using mass spectrometry. About 0.25 mg of ciliary proteins was analyzed in each sample. Proteins present in both control and experimental samples are highlighted in green.
(XLSX)

S5 Table Mass-spectrometry analysis of proteins biotinylated in cells expressing Ccdc113-HA-BirA* and BirA*-HA-Ccdc113. Proteins identified by only one peptide were not analyzed. Proteins present in both control and experimental samples are highlighted in green (C-terminal BirA*) or red (N-terminal BirA*).
(XLSX)

S6 Table Mass-spectrometry analysis of proteins biotinylated in cells expressing Ccdc96-HA-BirA* and BirA*-HA-Ccdc96. Proteins identified by only one peptide were not analyzed. Proteins present in both control and experimental samples are highlighted in green (C-terminal BirA*) or red (N-terminal BirA*).
(XLSX)

S7 Table Mass-spectrometry analysis of the ciliary proteins that co-immunoprecipitated with Ccdc113-3HA expressed at native levels. Proteins identified by only one peptide were not analyzed. Note that, besides Ccdc113, Ccdc96 was identified by the highest number of peptides.
(XLSX)

S8 Table List of primers used in this study. The nucleotide sequences recognized by the restriction endonucleases are in bold; the restriction sites introduced to screen for positive clones are in small letters.
(DOCX)

S9 Table Overview of the number of tomograms and particles used for the subtomogram averaging. (DOCX)

S10 Table Numerical data. (XLSX)

S1 Video High-speed video recording of beating cilia in wild-type cells. (AVI)

S2 Video High-speed video recording of beating cilia in *CCDC113-KO* cells. (AVI)

S3 Video High-speed video recording of beating cilia in *CCDC96-coDel* cells. (AVI)

S4 Video High-speed video recording of beating cilia in *DRC3-coDel* cells. (AVI)
