# Supplementary figures and images for: Ccdc113/Ccdc96 complex, a novel regulator of ciliary beating that connects radial spoke 3 to dynein g and the nexin link
Source: PLoS Genet. 2021 Mar 4;17(3):e1009388. doi: 10.1371/journal.pgen.1009388 (PMC7987202; doi:10.1371/journal.pgen.1009388)

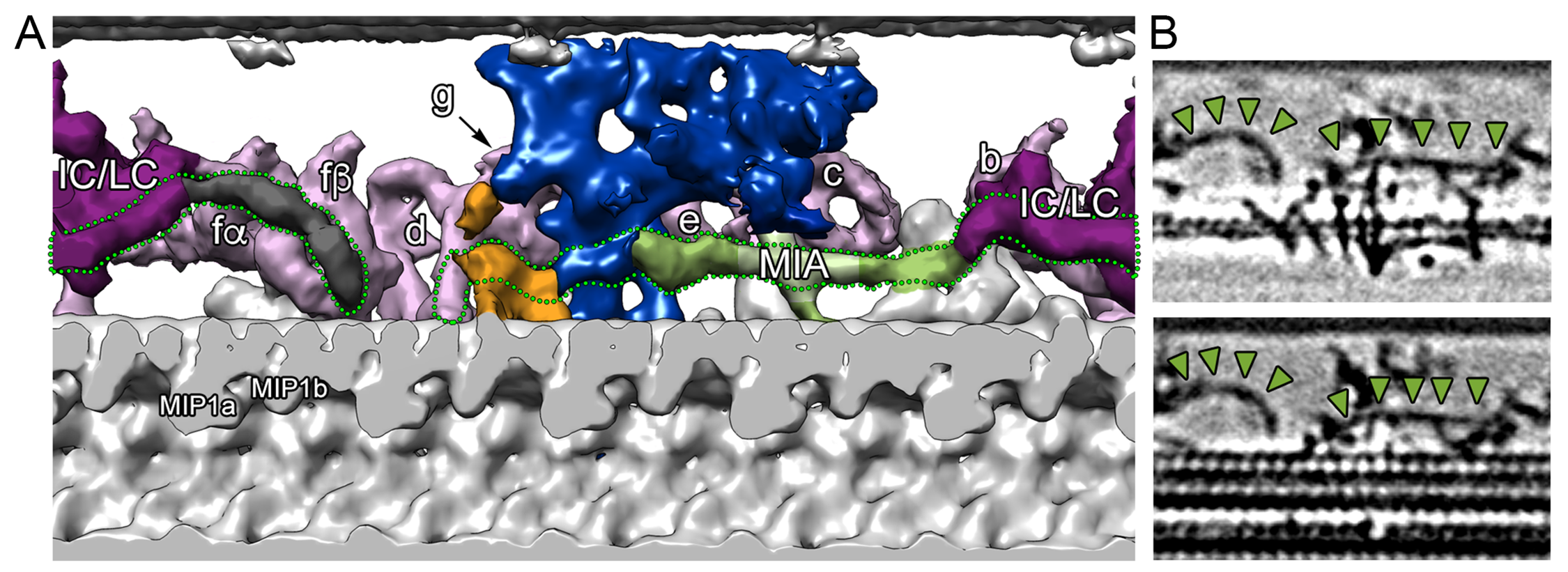

Supplement: S1 Fig — (A) A 96-nm linker (green dotted outlines) bridges structures throughout the whole 96-nm repeat, extending from the tail of IDA g (pink) to the heavy chain β of IDA I1/f (pink), going through the N-DRC (navy blue), MIA-like complex (light green) and IC/LC (purple) of the IDA I1/f. (B) Tomographic slices of 96-nm linker. (TIF) [file pgen.1009388.s001.tif]

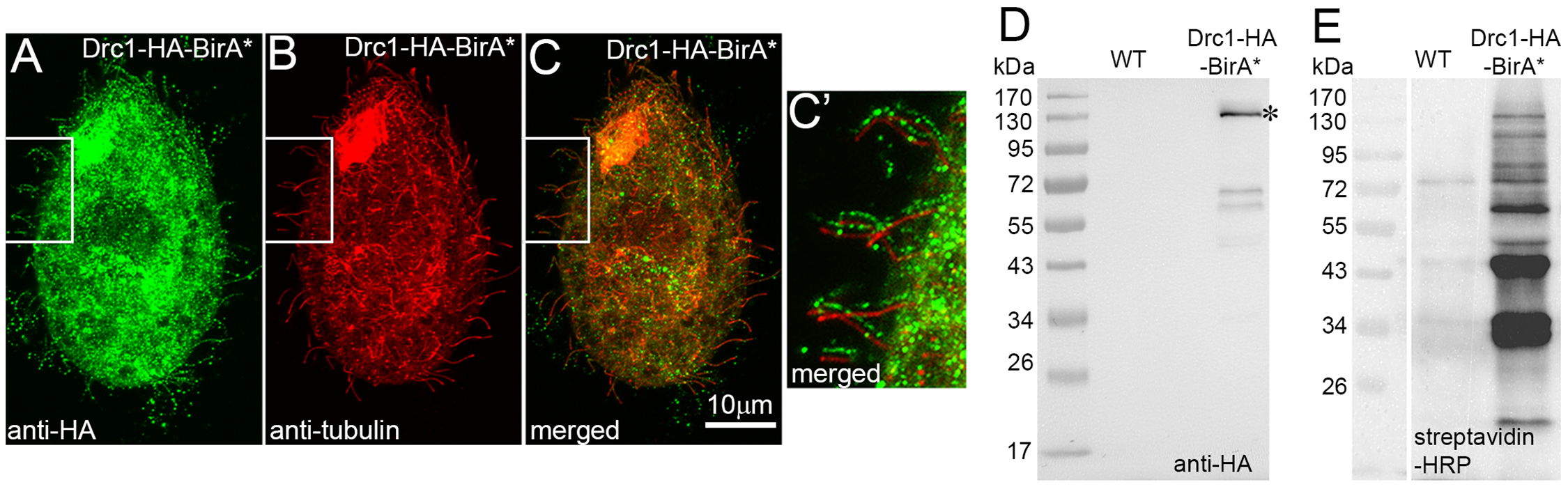

Supplement: S2 Fig — (A-C) Immunofluorescence confocal images of Tetrahymena cells expressing Drc1-HA-BirA* at the native level, double labeled with anti-HA (A) and anti-tubulin (B) antibodies. (C) Merged image of A and B. (C’) A magnified fragment of (C) indicated by a while inset. Note that red and green channels were slightly shifted to better visualize localization of Drc1-HA-BirA* in cilia. (D) Western blot of the ciliary proteins isolated either from wild-type cells (WT) or cells expressing Drc1-HA-BirA* under the control of a native promoter. A star indicates the position of the Drc1-HA-BirA* protein (~126 kDa). The additional, faster migrating bands are most likely partly degraded Drc1-HA-BirA* fusion protein. (E) Detection of the biotinylated proteins in cilia isolated from either WT cells or cells expressing Drc1-HA-BirA* at native levels grown in medium supplemented with biotin for 4 hrs. (TIF) [file pgen.1009388.s002.tif]

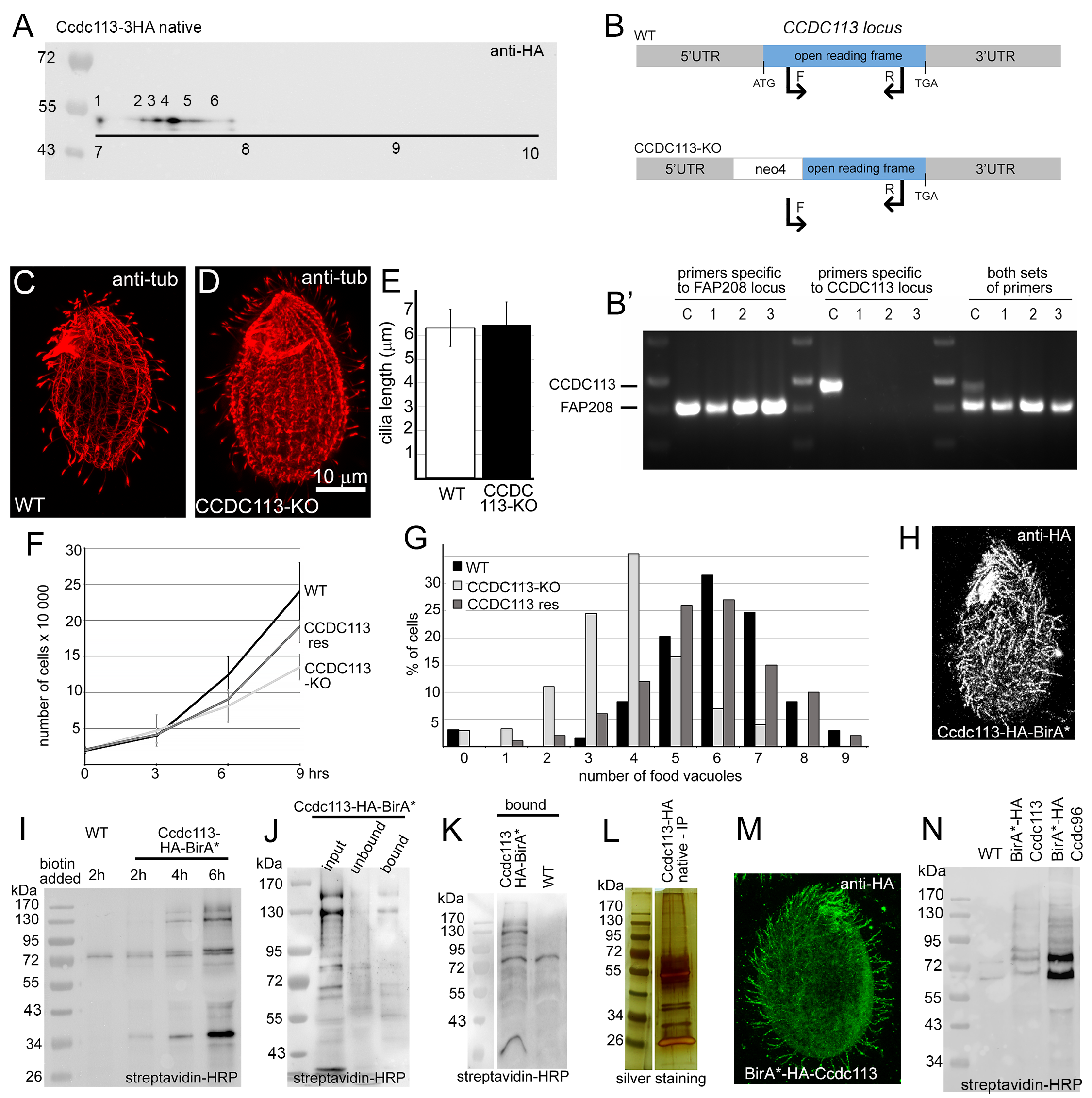

Supplement: S4 Fig — (A) Two-dimensional analyses of axonemal proteins (30 μg) purified from cells expressing Ccdc113-3HA under the control of the native promoter. Isoelectric focusing was performed using 7 cm 7–10 ready-strips. The theoretical calculated pI = 8.87 (https://web.expasy.org/compute_pi). Note that all isoforms are more acidic, suggesting posttranslational modification. (B, B’) Changes in the CCDC113 locus in engineered knockout cells. (B) A schematic representation of the CCDC113 locus in a wild-type (WT) and CCDC113-KO cells. Blue rectangles represent the CCDC113 open reading frame, grey rectangles represent 5’ and 3’ UTRs. A white rectangle marks the position of a neo4 cassette that replaced a fragment of the 5’UTR and the open reading frame. Arrows indicate the annealing positions of the primers used to test alteration in CCDC113 locus. (B’) PCR analysis of the CCDC113 locus showing that part of the CCDC113 gene is deleted. PCR amplification of a fragment of the unrelated FAP208 locus was performed to verify the quality of isolated genomic DNA. (C-E) Knockout of CCDC113 does not affect cilia assembly and cilia length. Immunofluorescence confocal images of WT (C) and CCDC113-KO cells (D) stained with anti-α-tubulin antibodies. Scale bar = 10 μm. (E) Graphical representation of cilia length measurements of WT (white bar, 6.36 μm +/- 0.61, n = 60) and CCDC113-KO (grey bar, 6.7 μm +/- 0.77, n = 60) cells. Bars represent standard deviation. (F-G) Expression of Ccdc113-3HA restores normal phagocytosis and proliferation rates. (F) Graphical representation of the proliferation rate of WT, CCDC113-KO and CCDC113-KO rescued cells. (G) Graphical representation of the efficiency of the formation of food vacuoles. Cells were grown in medium supplemented with India ink and the number of India ink-filled food vacuoles per cell was scored. (H) Immunofluorescence analyses showing that Ccdc113-HA-BirA* localizes in cilia. (I-K) Detection of the biotinylated proteins: (I) in cilia isolate [file pgen.1009388.s004.tif]

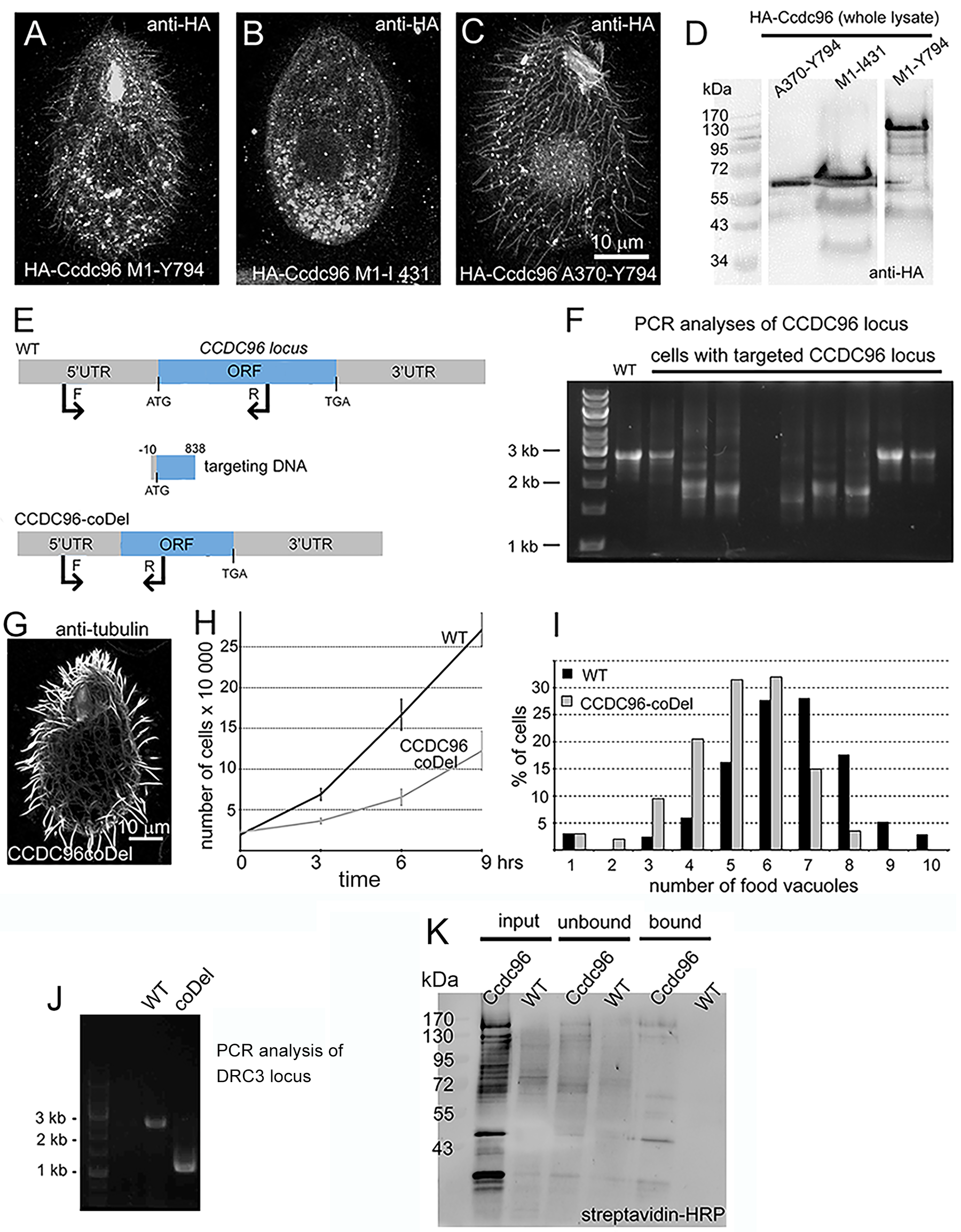

Supplement: S5 Fig — (A-C) Immunofluorescence confocal images of Tetrahymena cells overexpressing either HA-Ccdc96 full length protein (A), N-terminal fragment M1-I 431 (B), or C-terminal fragment A370—Y794 (C). Note that the C-terminal domain is indispensable and sufficient for protein ciliary localization. (D) Western blot of the total cell extract obtained from cells overexpressing truncations or full-length Ccdc96 protein. Note that Ccdc96 is prone to degradation in total extract from Tetrahymena cells. (E-F) Alteration in the CCDC96 locus in engineered CCDC96-coDel cells. (E) Schematic of the CCDC96 locus in wild-type (WT) and CCDC96 mutant cells obtained using the co-Deletion method. Blue rectangles represent the CCDC96 open reading frame, grey rectangles represent 5’ and 3’ UTRs. Arrows indicate the annealing positions of the primers used to analyze the extent of deletion in the CCDC96 locus. (F) PCR analysis of the CCDC96 locus in independently obtained deletion mutants with the indicated primers (see S5E) annealing about 1kb upstream and 1 kb downstream of the gene fragment (targeting DNA) inserted into the pMcoDel plasmid. Note that the PCR fragment amplified using WT genomic DNA as a template (control) is larger than PCR fragments amplified using genomic DNA isolated from mutants, indicating deletion of the fragment of the gene. (G) Lack of Ccdc96 does not affect cilia length. Staining of CCDC96-coDel cell with anti-α-tubulin antibodies (12G10) revealed that mutant cells assemble cilia of a similar length (6.2 μm +/- 0.54 (standard deviation), n = 50) to WT cells (6.36 μm +/- 0.61, n = 60). (H) Deletion of CCDC96 reduces proliferation rate. (I) Graphical representation of the efficiency of the formation of food vacuoles. On average (data from three independent experiments), WT cells formed 5.9 vacuoles (n = 300 cells), CCDC96-coDel mutants 4.8 vacuoles (n = 300) and CCDC96-coDel rescued cells 6.2 vacuoles (n = 300, not shown). (J) PCR analysis of the DRC3 locus with primers a [file pgen.1009388.s005.tif]

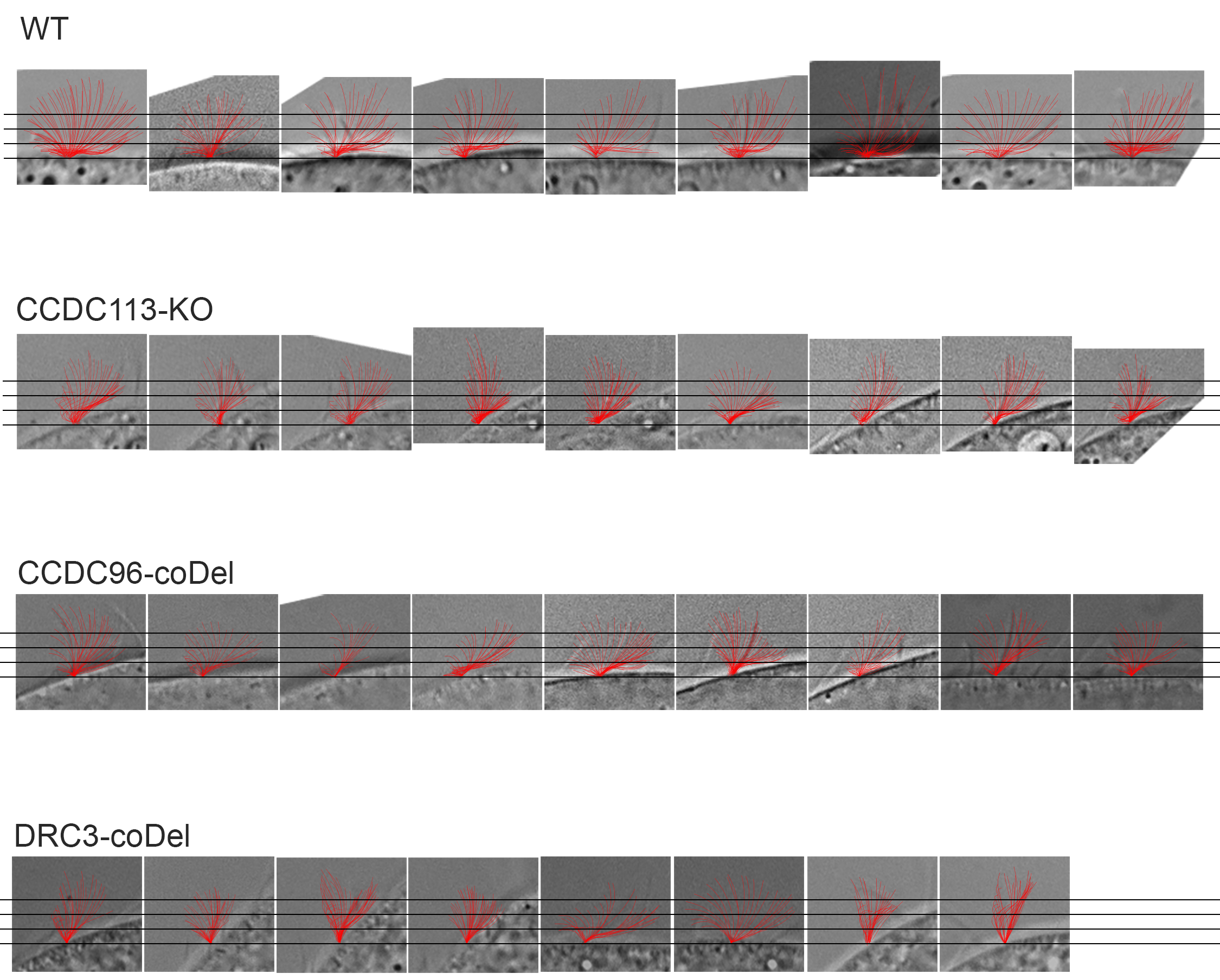

Supplement: S6 Fig — Additional analyses of the ciliary amplitude (schematic representation of all recorded consecutive positions of the cilium during the power and recovery stroke). (TIF) [file pgen.1009388.s006.tif]

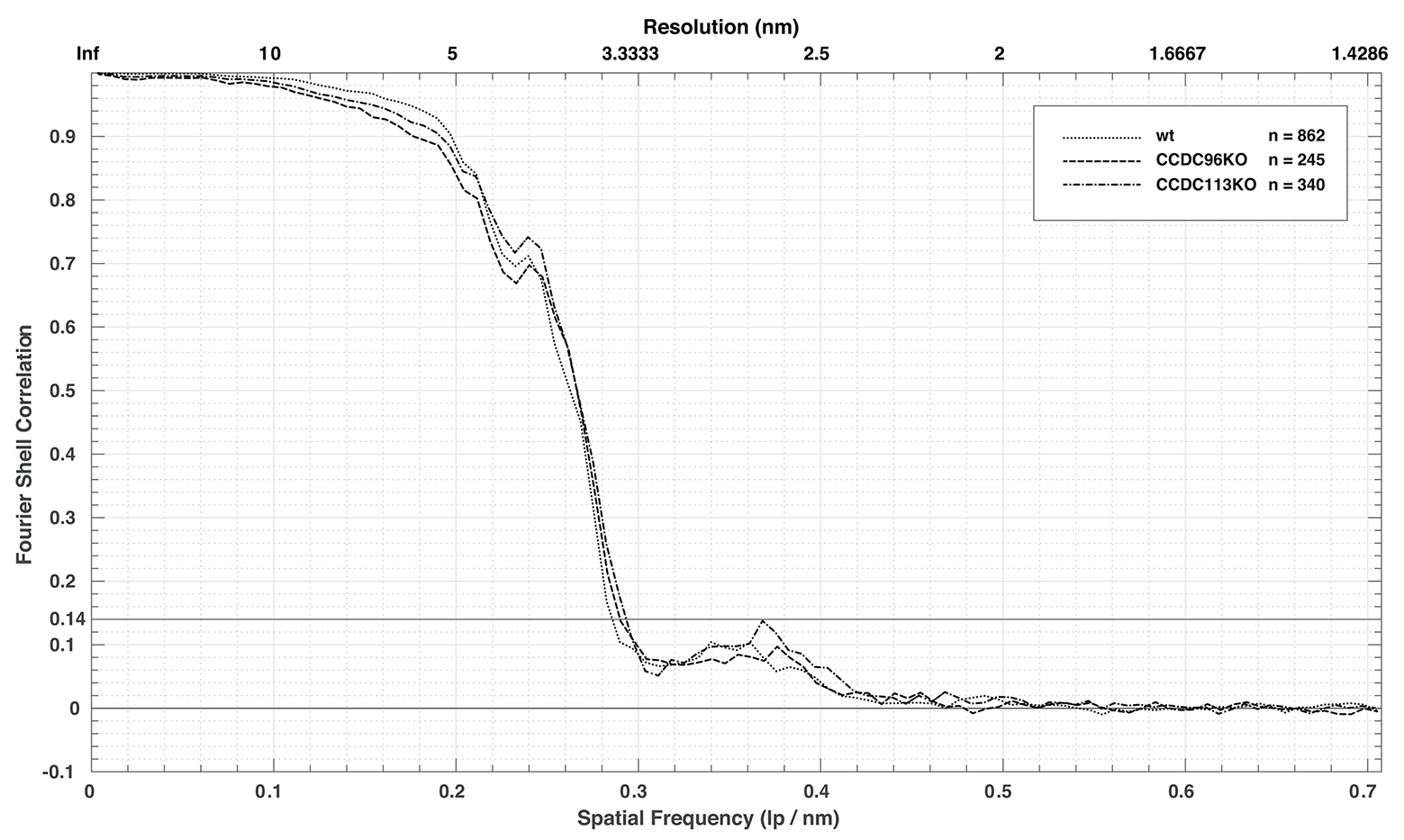

Supplement: S7 Fig — Assessment of structural measurements performed on averaged data of axonemal 96-nm repeats from the wild type (wt) and two types of mutant cells (CCDC113-KO, CCDC96-coDel) of Tetrahymena thermophila. Fourier Shell Correlation (FSC) curves of the average electron density map from Tetrahymena 96-nm repeats depicting the resolution associated with typical criteria (FSC = 0.143). (TIF) [file pgen.1009388.s007.tif]

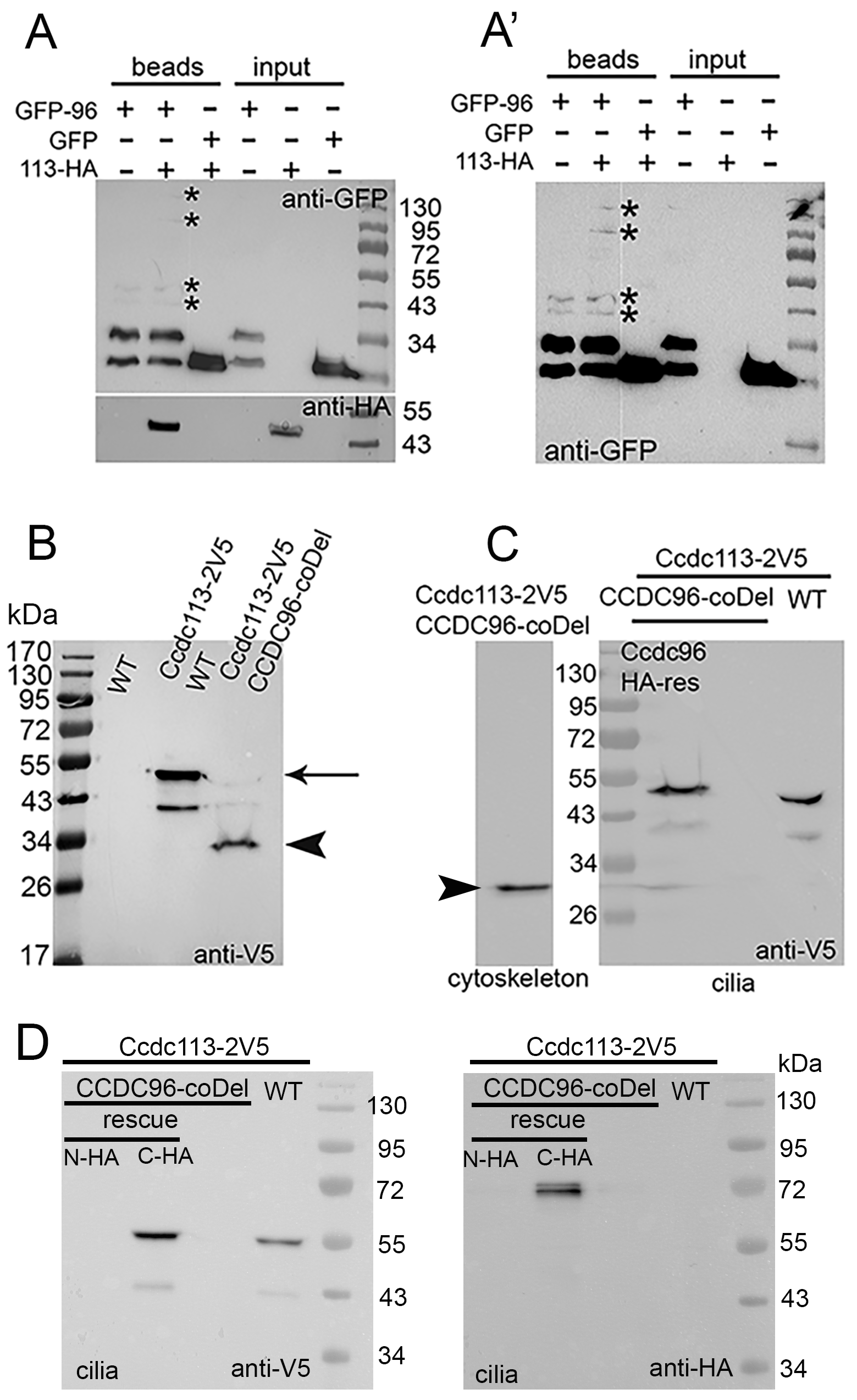

Supplement: S8 Fig — (A, A’) Cytoplasmic GFP-Ccdc96 but not GFP can pull down Ccdc113-HA. Note that overexpressed GFP-Ccdc96 present in the cytoplasm is prone to degradation (stars mark weakly visible non-degraded and partly degraded GFP-Ccdc96. (A’) A longer exposure of the same blot to better visualize less degraded forms of GFP-Ccdc96. (B, C) Western blot of the cytoskeletal (B and C, left panel) and ciliary (C, right panel) proteins isolated from WT cells and cells expressing Ccdc113-2V5 either in WT or CCDC96-coDel background, or in CCDC96-coDel mutants rescued with HA-Ccdc96 expression. Note that HA signal is not detected in WT cells (B) and that Ccdc113-3HA is partly degraded (arrow head) in cells lacking Ccdc96 (B and C, left panel). (D) Western blot of the ciliary proteins isolated from cells expressing Ccdc113-2V5 either in WT or CCDC96-coDel background, or in CCDC96-coDel mutants rescued with HA-tagged N-terminal fragment (M1-I431, named N-HA) or C-terminal fragment (A370-Y794, C-HA) of Ccdc96 protein. Note that Ccdc113-2V5 was detected in WT cells or cells rescued with the C-terminal fragment of Ccdc96 (blot to the left) but not with the N-terminal fragment that is not targeted to cilia (blot to the right). (TIF) [file pgen.1009388.s008.tif]

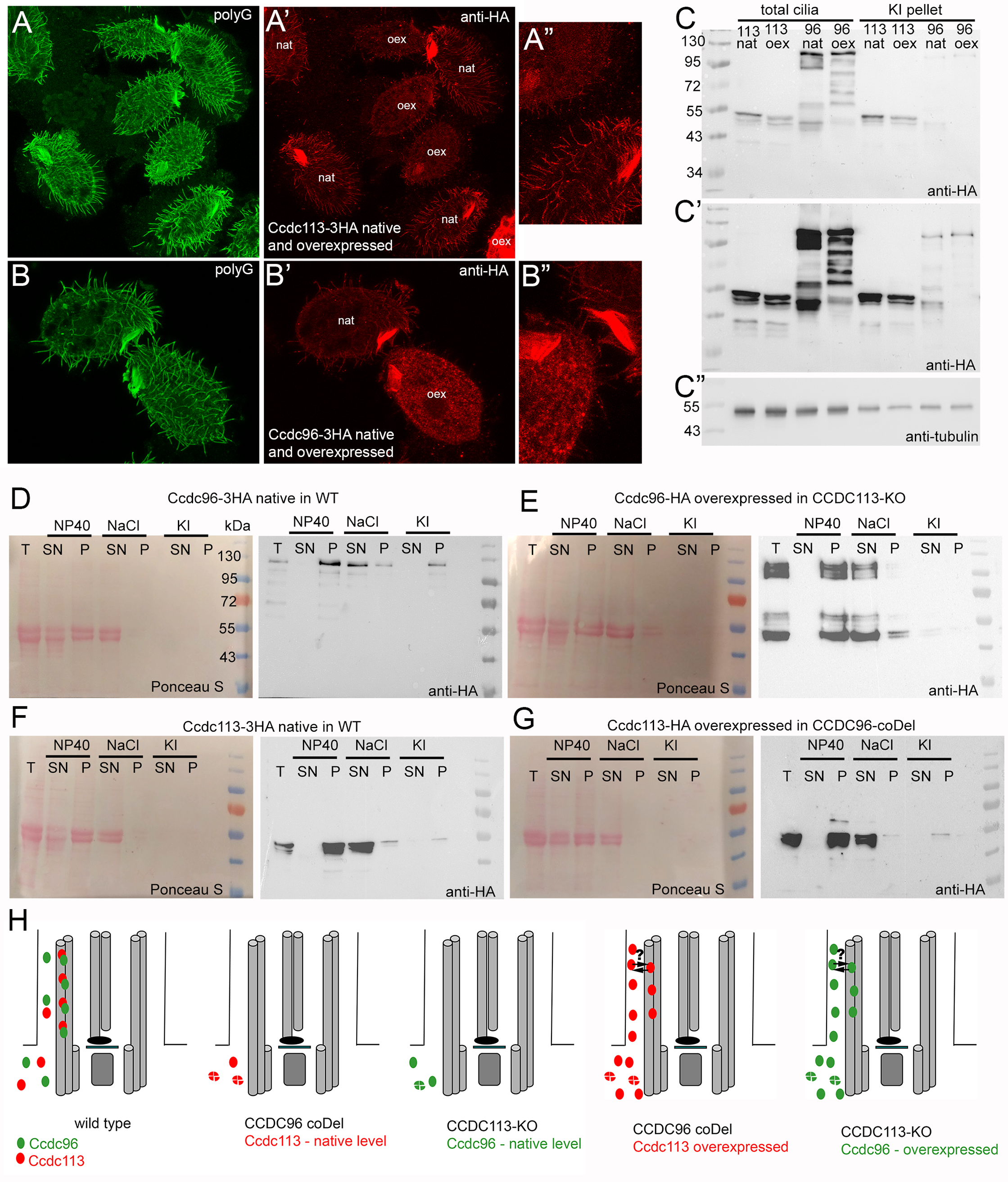

Supplement: S9 Fig — (A-B”) Comparison of the level of HA signal in cells either expressing HA-tagged Ccdc113 (A-A”) or Ccdc96 (B-B”) under the control of the respective native promoter in otherwise wild-type background (nat) or overexpressing these proteins (oex) in a mutant background (deletion of the partner proteins). Note only a weak HA-positive signal in cilia of knockout cells compared with wild-type cells expressing tagged proteins at the native level. (A, B) polyG (anti-polyglycylation antibody to visualize cilia), (A’, B’) anti-HA antibody, (A”, B”) enlarged fragments of A’ and B’, respectively. (C-C”) Western blot of the total ciliary proteins (lines 1–4) and axonemal pellet remaining after 0.5 M KI treatment (lines 5–8) showing the level of Ccdc113 and Ccdc96 proteins in cilia and amount of the proteins bound to the axoneme. Cilia were isolated from: wild-type cells expressing Ccdc113-3HA under the control of the native promoter (113 nat), CCDC96-coDel cells overexpressing Ccdc113-HA (113 oex), wild-type cells expressing Ccdc96-3HA under the control of the native promoter (96 nat), CCDC113-KO cells overexpressing Ccdc96-HA (96 oex), (C’) Longer exposure of a blot presented in (C). (C”) The level of tubulin in the analyzed samples (a loading control). (C, C’) Note slightly lower level of the proteins in samples obtained from knockout cells overexpressing partner protein (lines 6 and 8) compared to the wild-type cells expressing the same partner protein (lines 5 and 7) suggesting less effective binding of the protein to the axonemes. (D-G) Western blot analyses of total ciliary proteins (T) and ciliary proteins present in the cilia fractions (supernatant (SN) and pellet (P)) obtained after 1% NP-40 treatment, extraction of the axonemes from NP-40 pellet with the 0.6 M NaCl and extraction of the axonemes collected after NaCl treatment, with 0.5M KI (according [44]). Proteins were visualized using Ponceau S and HA-tagged proteins were detected using anti-HA antibodies. (H) A sch [file pgen.1009388.s009.tif]
