# Supplementary material for: Ccdc113/Ccdc96 complex, a novel regulator of ciliary beating that connects radial spoke 3 to dynein g and the nexin link
Source: PLoS Genet. 2021 Mar 4;17(3):e1009388. doi: 10.1371/journal.pgen.1009388 (PMC7987202; doi:10.1371/journal.pgen.1009388)

**CCDC113**


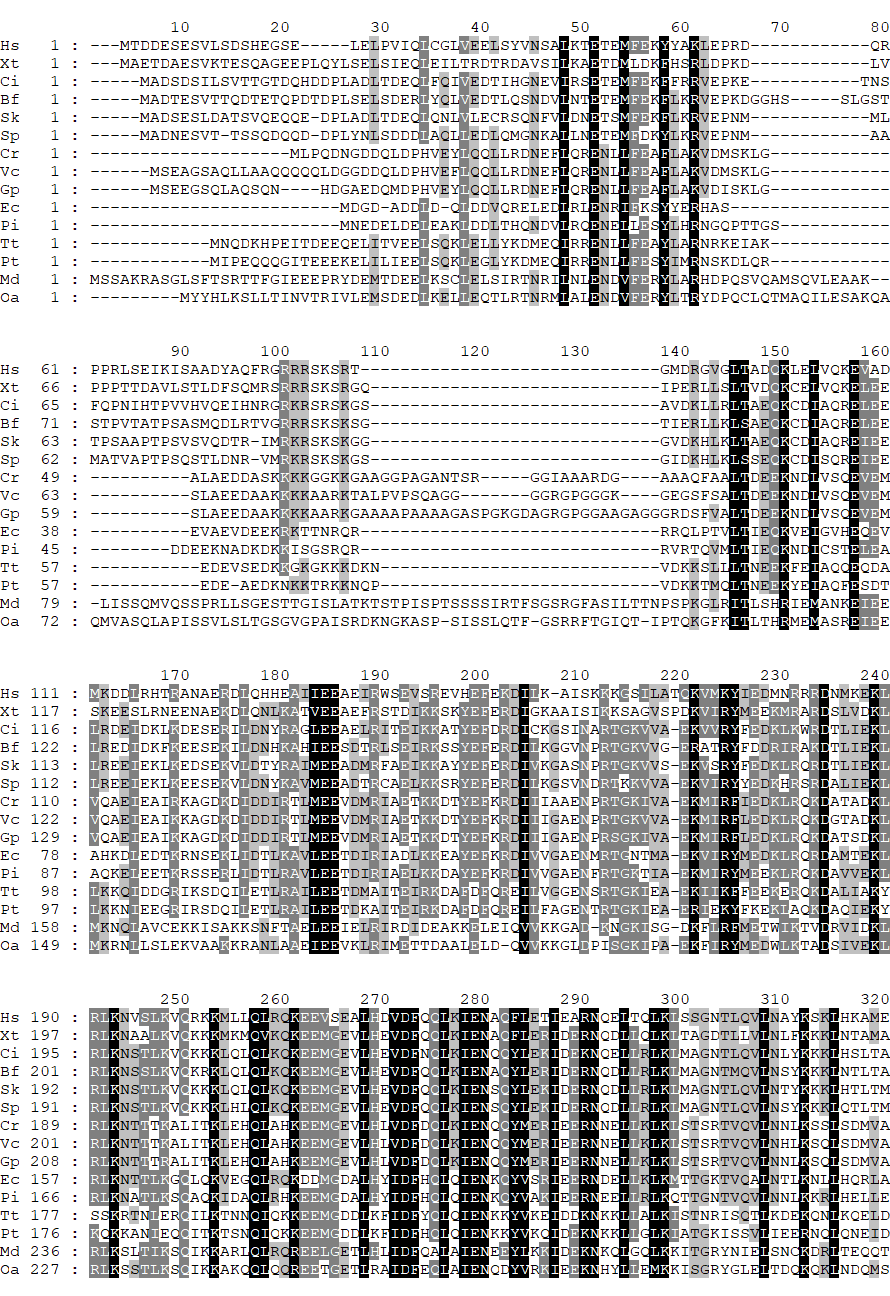


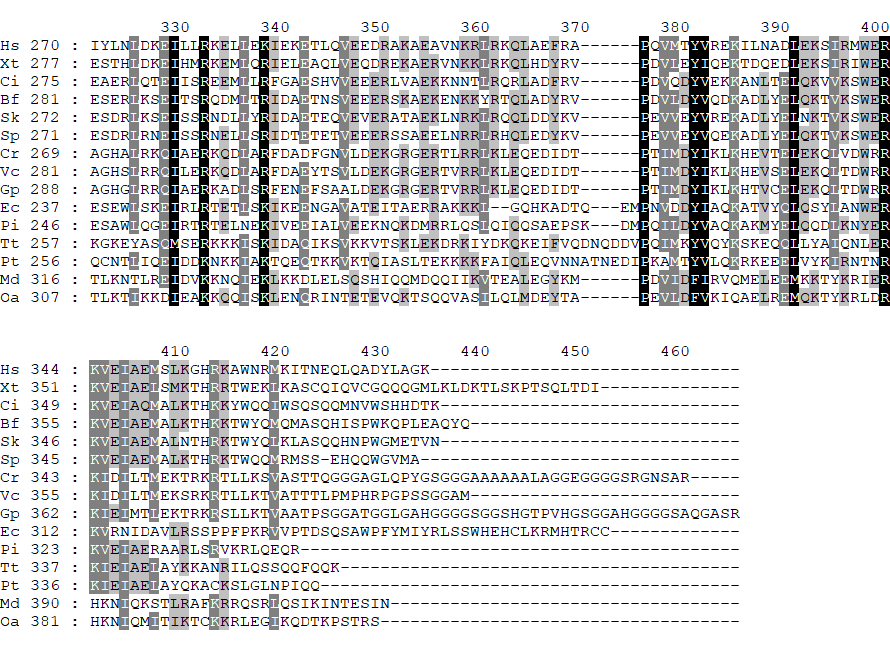


**Data obtained using COILS**

(https://embnet.vital-it.ch/software/COILS_form.html)


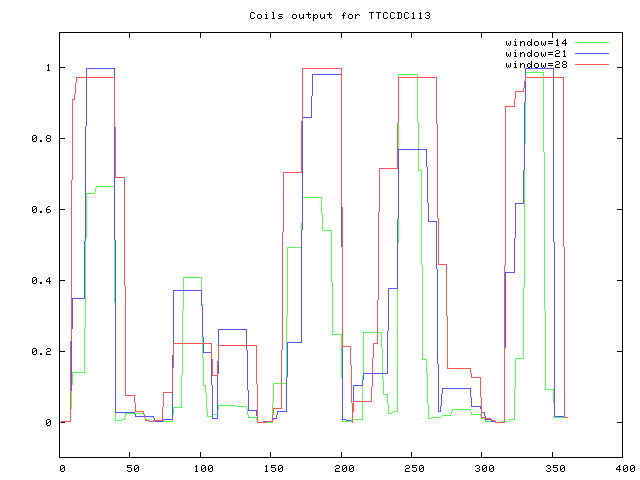


**CCDC113 - Phylogenetic tree**

**
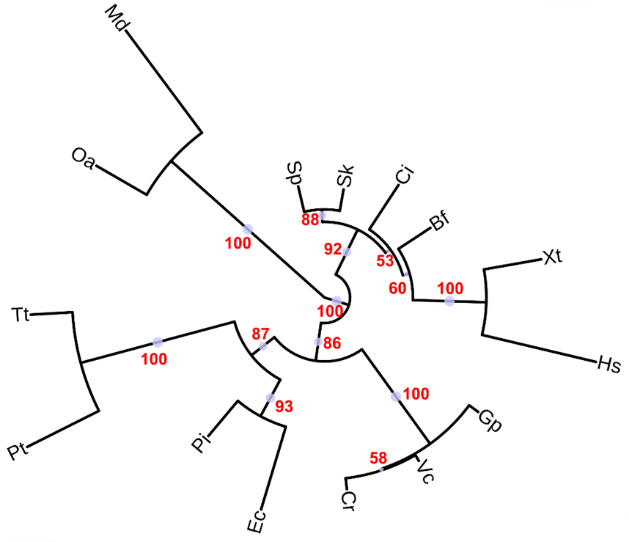
**

**CCDC96**


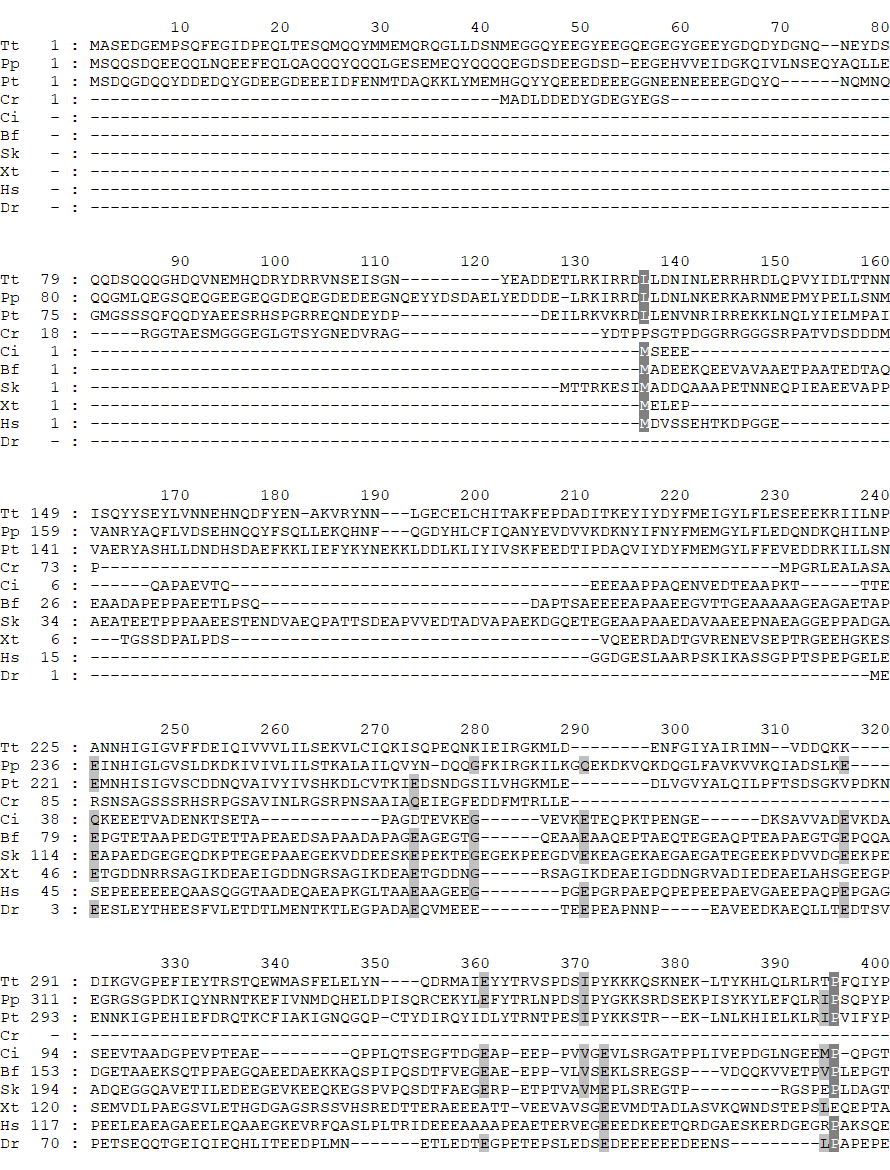


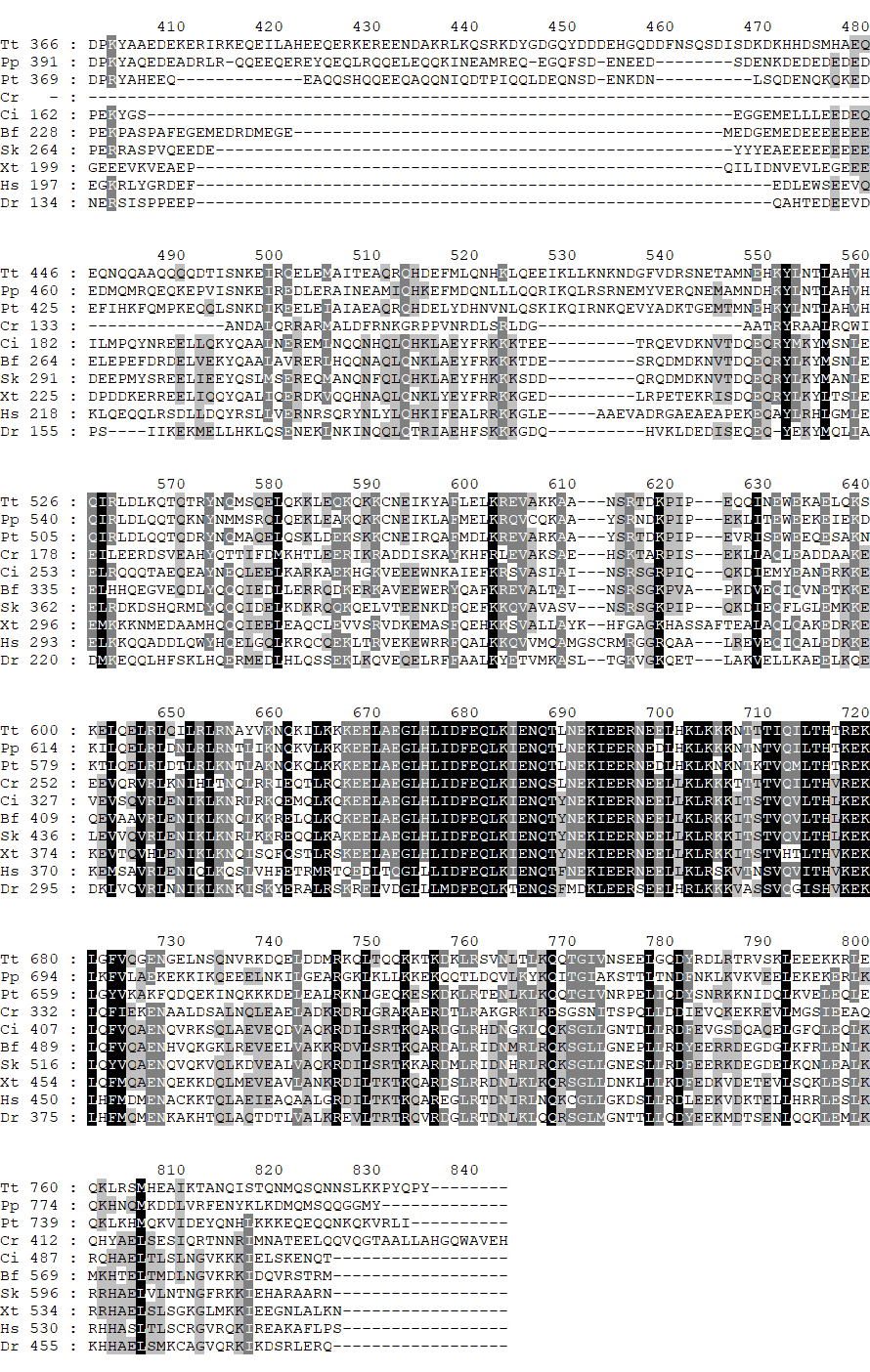


**Data obtained using COILS**


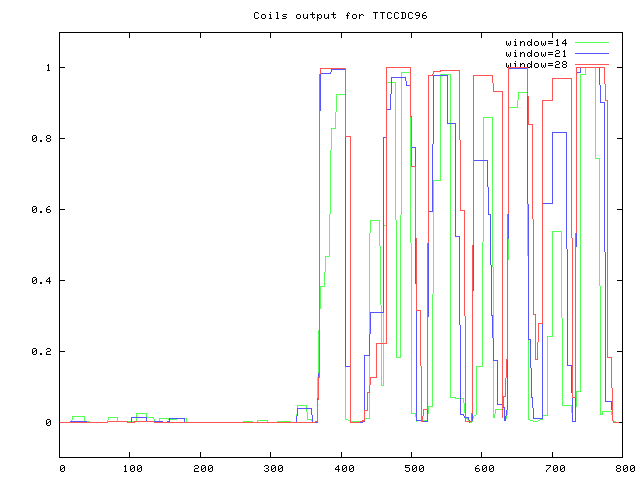


**CCDC96 - Phylogenetic tree**


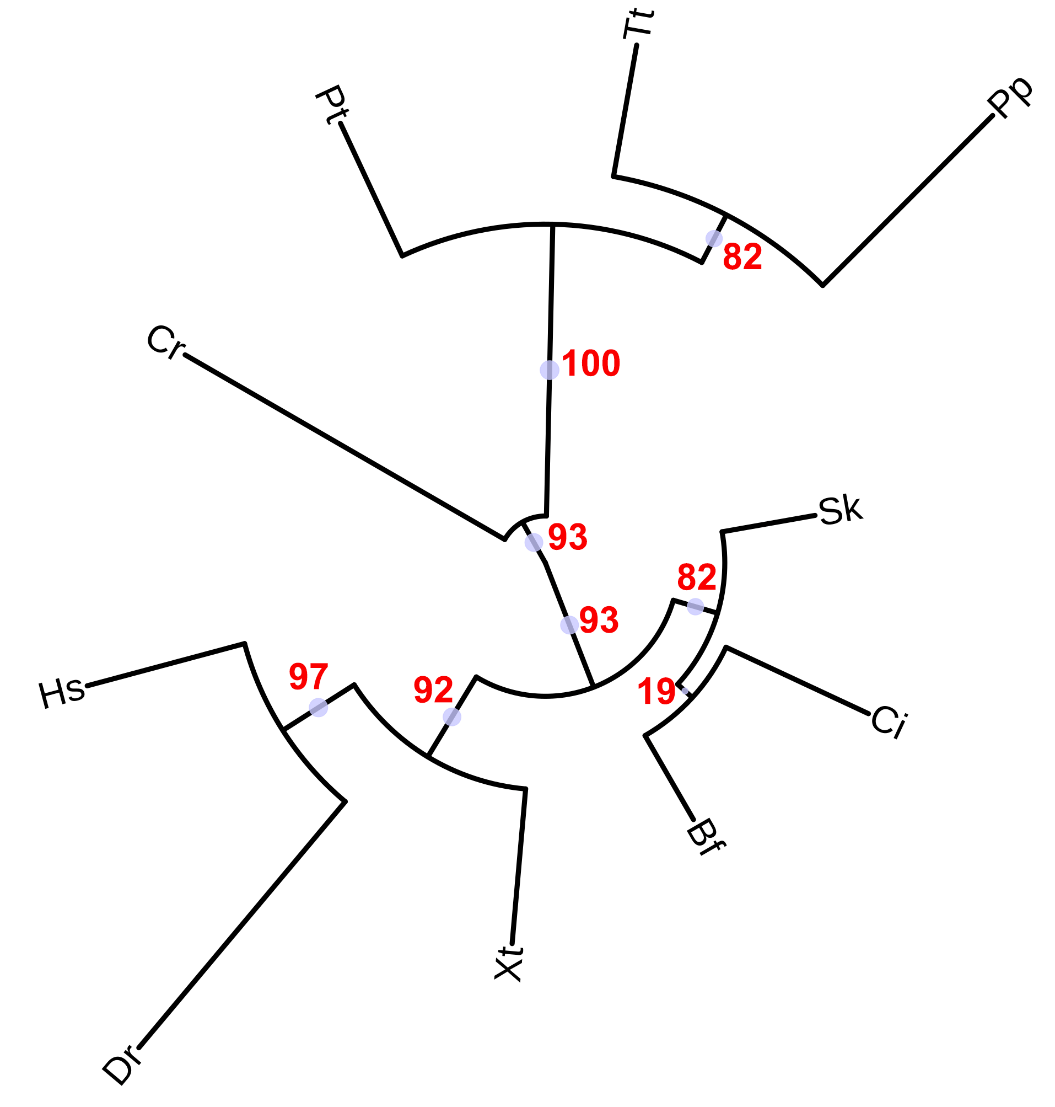

Supplement: S3 Fig — Ccdc96 and Ccdc113 homologs were obtained from the NCBI protein database using Blastp search and either human or Tetrahymena proteins as bait. Protein amino acid sequences were aligned using ClustalX2 software [66] and edited using SeaView [67]. The identical and similar amino acid residues were shaded using GeneDoc [68]. The phylogenetic tree was calculated (www.phylogeny.fr) [69–74] and the tree was drawn using iTOL (https://itol.embl.de) [75]. The branch support values are shown as %. The coiled-coil domains (blue bars) were predicted using SMART (http://smart.embl-heidelberg.de/) [77] and COILS (https://embnet.vital-it.ch/software/COILS_form.html) [78]. Ccdc113 orthologs used: Branchiostoma floridae (Bf, XP_002594168.1), Chlamydomonas reinhardtii (Cr, XP_001703742.1), Ciona intestinalis (Ci, XP_002125206.1), Ectocarpus siliculosus (Ec, CBJ30690.1), Gonium pectoral (Gp, KXZ50957.1), Homo sapiens (Hs, NP_054876.2), Microplitis demolitor (Md, XP_008557297.1), Orussus abietinus (Oa, XP_012276405.1), Paramecium tetraurelia (Pt, XP_001431423.1), Phytophthora infestans (Pi, XP_002997358.1), Saccoglossus kowalevskii (Sk, XP_002741623.1), Strongylocentrotus purpuratus (Sp, XP_785529.1), Tetrahymena thermophila (Tt, XP_001033462.1, TTHERM_00312810), Volvox carteri f. nagariensis (Vc, XP_002949615.1), Xenopus tropicalis (Xt, AAH89076.1). Ccdc96 orthologs used: Branchiostoma floridae (Bf, XP_002603613.1), Chlamydomonas reinhardtii (Cr, XP_001697427.1), Ciona intestinalis (Ci, XP_002126679.1), Danio rerio (Dr, NP_001122170.1), Homo sapiens (Hs, NP_699207.1), Paramecium tetraurelia (Pt, XP_001455440.1), Pseudocohnilembus persalinus (Pp, KRX11190.1), Saccoglossus kowalevskii (Sk, XP_002733290.1), Tetrahymena thermophila (Tt, XP_001032676.1), Xenopus tropicalis (Xt, XP_002938310.2). (DOCX) [file pgen.1009388.s003.docx]
