# Supplementary material for: Ccdc113/Ccdc96 complex, a novel regulator of ciliary beating that connects radial spoke 3 to dynein g and the nexin link
Source: PLoS Genet. 2021 Mar 4;17(3):e1009388. doi: 10.1371/journal.pgen.1009388 (PMC7987202; doi:10.1371/journal.pgen.1009388)
Supplement: S8 Table — The nucleotide sequences recognized by the restriction endonucleases are in bold; the restriction sites introduced to screen for positive clones are in small letters. (DOCX) [file pgen.1009388.s017.docx]

**Table S8**

List of primers used in this study. The nucleotide sequences recognized by the restriction endonucleases are in bold (restriction enzyme name is a part of the primer name), the restriction sites introduced to screen for positive clones are in small letters, start and stop codon are underlined.

| **Primer name** | **Nucleotide Sequence** | **PCR product and Transgene** |
| --- | --- | --- |
| **CCDC113** | | |
| **Overexpression** | | |
| CCDC113-OEX-F-MluI | ATAT **ACGCGT** C ATGAATTAAGATAAGCATCCTG | entire open reading frame  transgene enabling overexpression of Ccdc113 with N- or C- terminal tag. |
| CCDC113-OEX-R-BamHI (with TGA) | ATAT **GGATCC** GAATAGGCTTGATGAATAATCTTA (primer within 3’UTR) |  |
| CCDC113-Nat-R-BamHI (without TGA) | ATT **GGATCC** TTTCTATTAGAATTATTAACTAGATTATAAGATTCTATTT (underline letter indicate silent mutation) |  |
| **Native locus expression** | | |
| CCDC113-Nat-F-MluI | AATT **ACGCGT** TATGACAGACATGGCAATCACTG | 2 kb of a C-terminal fragment of the open reading frame  1.6 kb of a 3’UTR  transgene enabling expression of Ccdc113 with C- tag in native locus |
| CCDC113-Nat-R-BamHI  (without TGA) | ATT **GGATCC** TTTCTATTAGAATTATTAACTAGATTATAAGATTCTATTT |  |
| CCDC113-3UTR-F-PstI | AATT **CTGCAG** GTTTTCAGCTAAAA ATCTAAGGATCG |  |
| CCDC113-3UTR-R-XhoI | AATT **CTCGAG** GCAAGAGTCCAGATCCAG |  |
| **Gene knockout** | | |
| CCDC113-KO-5-F-ApaI | AATT **GGGCCC** GATTGGAGTAGAAAGAGAGAGATACTTG | 1.9 kb fragment encompassing 1.5 kb of the 5’UTR and 0.4 kb of the open reading frame, upstream to the deleted gene fragment (0.75 kb)  1.7 kb fragment encompassing 1.6 kb of the open reading frame and 0.1 kb of 3’UTR downstream to the deleted gene fragment  Transgene to knockout *CCDC113* gene |
| CCDC113-KO-5-R-SmaI | AATT **CCCGGG** GCAATTTCCTTCCTGTTTCTCGCC |  |
| CCDC113-KO-3-F-PstI | AATT **CTGCAG** TGGGAGATGACCTGAAATTCATTG |  |
| CCDC113-KO-3-R-SacII | AATT **CCGCGG** GCAAATTTGTGAAGCGAGCTGTG |  |
| CCDC113-OEX-F-MluI (check) | ATAT **ACGCGT** CATGAATTAAGATAAGCATCCTG | Primers used to verify if 0.75 kb fragment of *CCDC113* gene was deleted in constructed knockout cells |
| CCDC113-Del-R  (check) | TAGAACTGGTAAGATTGAAGCAGA |  |
|  | | |
| **CCDC96** | | |
| **Overexpression and domain analyses** | | |
| CCDC96-OEX-F-MluI | ATAT **ACGCGT** T ATGGCAAGTGAAGATGGTG | entire open reading frame  transgene enabling overexpression of Ccdc96 with N-terminal tag. |
| CCDC96-OEX-R-BamHI | ATAT **GGATCC** TCA ATAAGGCTAATATGGTTTTTTTAATGA |  |
| CCDC96-OEX-N-domain-R-BamHI | ATAT **GGATCC** TCATATATCAGACTATGAGTTGAAATCG | fragments of the open reading frame  used in combination of the overexpression primers |
| CCDC96-OEX-C-domain-F-MluI | ATAT **ACGCGT** T ATGGCTGCTGAAGATGAAAAAGAAAG |  |
| **Native locus expression** | | |
| CCDC96-Nat-F-MluI | AATT **ACGCGT** CGATTTCAACTCATAGTCTG ATATATC | 1.3 kb of a C-terminal fragment of the open reading frame  1.3 kb of a 3’UTR  transgene enabling expression of Ccdc96 with C- tag in native locus. |
| CCDC96-Nat-R-BamHI  (without TGA) | AATT **GGATCC** ATAAGGCTAATATGGTTTTTTTAATGAG |  |
| CCDC96-3UTR-F-PstI | AATT **CTGCAG** GTATGCTTGATACATTATCGCAC |  |
| CCDC96-3UTR-R-XhoI | AATT **CTCGAG** GAAGGTTTTATGGAATATATTATGCC |  |
| **Gene knockout (coDel)** | | |
| CCDC96-coDel-F | CAGTTCTCATCAAGTTGTAATGCTAAAAT**GCGGCCGC**TTTATACAAAATGGCAAGCGAAGATGG | 0.8 kb of the open reading frame  used to prepare transgene enabling gene deletion using coDel approach |
| CCDC96-coDel-R | GGACTCTTTATTGTTATCATCTTATGACC**GCGGCCGC**ATATATTCTTTAGTGATATCTGCATCTGGC |  |
| CCDC96-Check-F-MluI | AATT **ACGCGT** GAATATAGACAGCATGTTCCTTCAATT | Primers used to verify deletion of the fragment of *CCDC96* gene |
| CCDC96-Check-R-XhoI | AATT **CTCGAG** GTTCATTGCAGTTTCATTAGACCTATC |  |
|  | | |
| **FAP57A** | | |
| **Native locus expression** | | |
| FAP57-Nat-F-MluI | AATT **ACGCGT** G ATGGACAGAAGAAACGACTATG | 1.5 kb of a C-terminal fragment of the open reading frame  1.3 kb of a 3’UTR  transgene enabling expression of FAP57A with C- tag in native locus. |
| FAP57-Nat-R-BamHI  (without TGA) | AATT **GGATCC** ATTGTCTTCGTCATCGTCTTACTG |  |
| FAP57-3UTR-F-PstI | AAT **CTGCAG** GTTAATTGATTCTAC |  |
| FAP57-3UTR-R-XhoI | AATT **CTCGAG** TGGCGACTATTTGCTTCTTC |  |
|  | | |
| **DRC3** | | |
| DRC3-coDel-F | CAGTTCTCATCAAGTTGTAATGCTAAAAT**GCGGCCGC**ctcgagCAGTTGATCCAAGAGTCATCAATG | 0.85 kb of the open reading frame  used to prepare transgene enabling gene deletion using coDel approach |
| DRC3-coDel-R | GGACTCTTTATTGTTATCATCTTATGACC**GCGGCCGC**TTTTCTTTGTCTTTTGTGAATGGATTTC |  |
| DRC3-MluI-  Check-F | AATT **ACGCGT** GATTTCAGATGCCTTCAGAAGTG | Primers used to verify deletion of the fragment of *DRC3* gene |
| DRC3-XhoI-Check-R | AATT **CTCGAG** TCTAGTTATTACTACTGGGTACTGTTG |  |
|  | | |
| **Primers used to construct a transgene for expression in BTU2 locus** | | |
| BTU2-Promoter- SacI- F | AATT **GAGCTC** GAATACCAATTAAGGGTAGTACTG | 0.75 kb of BTU2 promoter |
| BTU2-Promoter-MluI-R | CATA**ACGCGT**GCCATCTTTTTTAAGTTATTTTTTTAGGG |  |
| BTU2-3UTR-PstI-F | AATT **CTGCAG** CGAAGAAGAAGAAGGTGAAAACTG | 1.5 kb of 3’UTR of BTU2 |
| BTU2-3UTR-XhoI-SacII-R | AATT**CCGCGGCTCGAG**GATAATATATTTAGATTCCCAAAATGAATGAATG |  |
