# Supplementary material for: Ccdc113/Ccdc96 complex, a novel regulator of ciliary beating that connects radial spoke 3 to dynein g and the nexin link
Source: PLoS Genet. 2021 Mar 4;17(3):e1009388. doi: 10.1371/journal.pgen.1009388 (PMC7987202; doi:10.1371/journal.pgen.1009388)
Supplement: S9 Table — (DOCX) [file pgen.1009388.s018.docx]

**Table S9**

Overview of the number of tomograms and particles used for the subtomogram averaging

| **Cell type** | **Number of tomograms** | **Number of particles** |
| --- | --- | --- |
| **Cu428** | 8 | 1725 |
| **CCDC113KO** | 3 | 689 |
| **CCDC96KO** | 3 | 499 |
